# Lipoprotein-associated phospholipase A2 (Lp-PLA2): a key hub linking lipid metabolism and immune inflammation

**DOI:** 10.3389/fimmu.2026.1705738

**Published:** 2026-01-26

**Authors:** Dandan Li, Yuanbo Qian, Li Wan, Kaixin Zhang, Lifeng Song, Xianjing Zhang, Xiaorong Yang

**Affiliations:** Affiliated Hospital of Zunyi Medical University, Zunyi, Guizhou, China

**Keywords:** biomarkers, immune regulation, lipid metabolism, Lp-PLA2, therapeutic targets

## Abstract

Lipoprotein-associated phospholipase A2 (Lp-PLA2), also known as phospholipase A2 group VII (PLA2G7), is an enzyme that serves as a critical nexus between lipid metabolism and immune regulation. It exhibits dual and context-dependent functions by hydrolyzing platelet-activating factor (PAF) and oxidized low-density lipoprotein (oxLDL). The degradation of PAF results in the production of Lysoplatelet activating factor (LysoPAF), which attenuates inflammatory signaling. In contrast, the hydrolysis of oxLDL generates lysophosphatidylcholine (LysoPC) and oxidized fatty acids (oxFA), which exacerbate vascular inflammation, promote macrophage M1 polarization, and inhibit CD8^+^ T cell activity. Through these pathways, Lp-PLA2 is implicated in a range of diseases, including atherosclerosis, diabetes, Alzheimer’s disease, cancer, autoimmune disorders, and inflammation associated with infections. Despite extensive pharmacological interventions targeting this enzyme, clinical outcomes have been inconsistent, reflecting its complex roles across various pathophysiological contexts. This review synthesizes current knowledge on the mechanisms of Lp-PLA2, its associations with diseases, and its therapeutic implications, emphasizing its potential as both a biomarker and a therapeutic target at the intersection of lipid metabolism and immune response.

## 1 Introduction

The phospholipase A2(PLA2) enzyme family constitutes a critical group of enzymes integral to lipid metabolism and the regulation of inflammatory processes. These enzymes catalyze the cleavage of membrane phospholipids, leading to the release of fatty acids and lysophospholipids, without inferring any specific bioactivities of the liberated products (1). This activity subsequently influences the synthesis of a range of bioactive lipid mediators (2).

**Figure 1:**
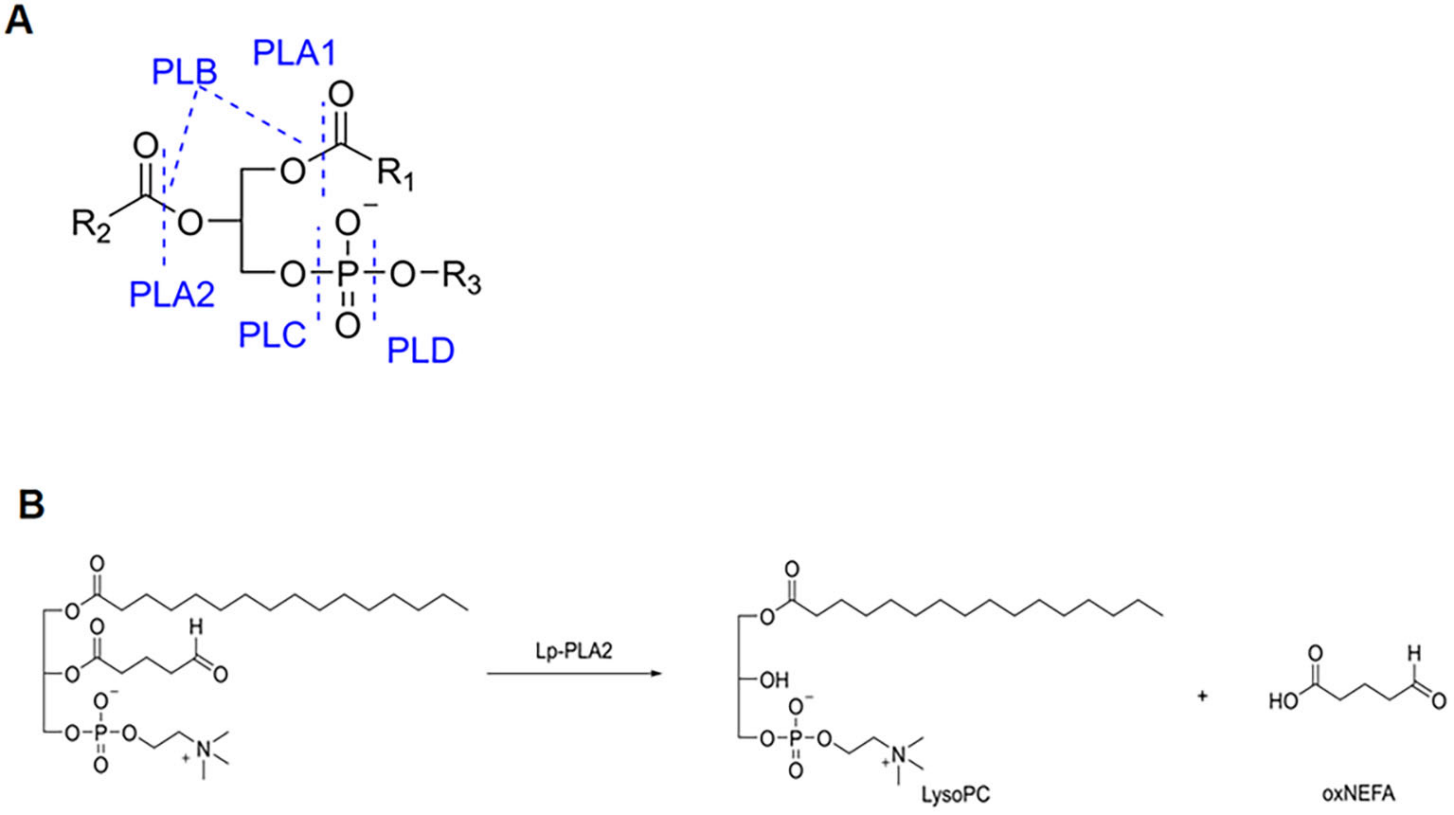
Schematic of phospholipase cleavage sites and oxidative phospholipolysis mediated by Lp-PLA2. **(A)** Schematic diagram illustrating the cleavage sites of different classes of phospholipases in phospholipid molecules. Based on the position of the hydrolyzed bond, phospholipases are classified into phospholipase A1 (PLA1), phospholipase A2 (PLA2), phospholipase B (PLB), phospholipase C (PLC), and phospholipase D (PLD). PLA1 and PLA2 hydrolyze the sn-1 and sn-2 ester bonds, respectively; PLB can hydrolyze both ester bonds; whereas PLC and PLD cleave distinct phosphodiester bonds, generating different lipid products. **(B)** Hydrolysis of oxidized phosphatidylcholine by Lp-PLA2. A representative oxidized phosphatidylcholine species, POVPC is hydrolyzed by Lp-PLA_2_ at the sn-2 ester bond, yielding LysoPC and an oxNEFA. This reaction represents a key biochemical step linking lipid oxidation to downstream inflammatory signaling.

**Figure 2:**
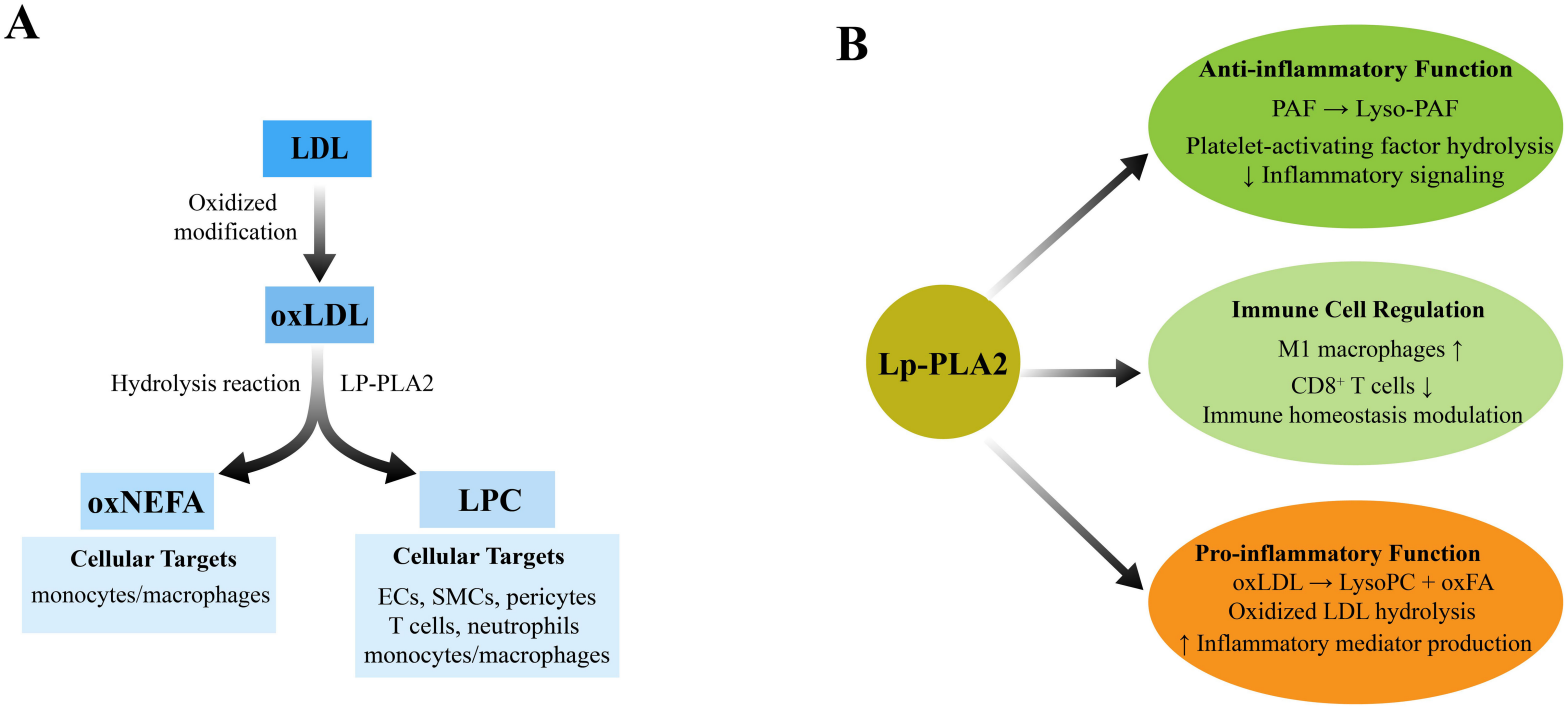
Schematic diagram of Lp-PLA2-mediated bidirectional metabolic pathways: **(A)** Lp-PLA2 catalyzes the hydrolysis of the sn-2 ester bond of oxidized phospholipids in oxLDL, yielding LysoPC and oxFA. LysoPC and oxFA act as pro-inflammatory factors, inducing endothelial cell activation and promoting the expression of adhesion molecules and chemokines (e.g., VCAM-1, ICAM-1, MCP-1), thereby amplifying inflammatory responses. **(B)** Lp-PLA2 catalyzes the hydrolysis of the sn-2 ester bond in PAF, generating LysoPAF. This deactivates PAF’s proinflammatory activity and initiates anti-inflammatory signaling pathways. LysoPAF activates the adenylate cyclase–cAMP–PKA signaling cascade, inducing immunosuppression and reducing the production of inflammatory cytokines.

These lipid mediators influence immune signaling, inflammation, and cellular homeostasis. Among the PLA2 family, Lp-PLA2—encoded by the PLA2G7 gene and historically known as platelet-activating factor acetyl hydrolase (PAF-AH)—is of particular importance. Lp-PLA2 is mainly secreted by macrophages and circulates in plasma bound to lipoproteins such as low-density lipoprotein (LDL) and high-density lipoprotein (HDL) (1, 3). It catalyzes the breakdown of oxidized phospholipids within oxLDL, especially oxidized phosphatidylcholines(oxPCs), producing two signaling molecules: LysoPC and oxidized non-esterified fatty acids (oxNEFA) (1). OxPCs comprise a heterogeneous group of lipid species. Among them, 1-palmitoyl-2-(5-oxovaleroyl)-sn-glycero-3-phosphocholine (POVPC) is one of the most extensively studied fragmented oxPCs and is commonly used as a representative substrate for Lp-PLA_2_ in mechanistic studies. As shown in **Figure 1**, Lp-PLA2 hydrolyzes oxidized phospholipids at the sn-2 position, yielding LysoPC and oxNEFA. This reaction constitutes the core biochemical basis for its role in inflammation regulation. LysoPC is the principal mediator of the proinflammatory effects associated with Lp-PLA2. It exerts its effects on a variety of cell types, including endothelial cells (EC), smooth muscle cells (SMC), monocytes/macrophages, T cells, and neutrophils. Lp-PLA2 can also exert a certain anti-inflammatory effect by degrading PAF, thus showing a typical “double-edged sword effect” in inflammatory regulation. LysoPC not only modulates cellular activities but also regulates the recruitment of inflammatory cells and modifies the functional responses of endothelial and smooth muscle cells. Additionally, it is involved in the induction of oxidative stress responses and the activation of immune response mechanisms (4). Numerous review articles have previously examined various aspects of Lp-PLA2, encompassing its structural characteristics, genetic underpinnings, biological functions, involvement in disease pathogenesis, and developments in specific inhibitors. Moreover, Lp-PLA2 has been extensively examined as a member of the phospholipase A2 superfamily, also known as PAF-AH (3, 5–9). Empirical evidence supports the pivotal role of Lp-PLA2 in a range of metabolic and inflammatory diseases, including atherosclerosis, diabetes, and autoimmune disorders (10–12). Consequently, Lp-PLA2 is integral not only to lipid metabolism but also to critical regulatory functions at the interface of innate and adaptive immunity (13). The enzymatic activity of PLA2G7 is influenced by several factors, such as genetic polymorphisms, hormonal levels, and external factors including pharmacological agents and dietary components (14). Research indicates that this enzyme is markedly expressed in various inflammatory and immune-related diseases, and it is widely acknowledged as a biomarker for inflammatory conditions, such as cardiovascular disease and chronic obstructive pulmonary disease (COPD) (15). Moreover, within the tumor microenvironment, phospholipase A2G7 facilitates the progression of cancer cachexia by modulating lipid metabolism, with its expression levels significantly correlating with patient weight loss and immunosuppression (16). Recently, the therapeutic potential of targeting phospholipase A2G7 has attracted considerable interest. For example, its specific inhibitor, darapladib has shown substantial promise in preclinical studies by enhancing antitumor immune responses through the restructuring of the lipid metabolic microenvironment, promoting ferroptosis, and regulating immune cell function (16). Consequently, advancing our understanding of Lp-PLA2’s role within the lipid-immune crosstalk network not only elucidates the pathophysiological basis of various diseases but also offers critical insights for the development of innovative immunometabolic intervention strategies. Due to its double-edged sword mechanism of action, this study first systematically summarizes the metabolic and immune interaction mechanisms of Lp-PLA2, as shown in **Figure 2**. The dual roles of Lp-PLA2 in pro-inflammatory versus anti-inflammatory lipid metabolism are summarized in **Figure 2**, which depicts the distinct reaction branches for oxLDL-derived oxidized phospholipids and PAF. Having established the dual functional nature of Lp-PLA2, we next discuss its structural features that underlie its substrate specificity and biological function.

## 2 Molecular and biological basis of Lp-PLA2

### 2.1 Structural features of human Lp-PLA2

#### 2.1.1 Domains and folding of human Lp-PLA2

Lp-PLA2 is a secreted enzyme characterized by an N-terminal sequence of 17 amino acid residues (Met1–Ala17), which potentially forms a hydrophobic signal peptide (17). The endogenous protein displays N-terminal heterogeneity, with initiation sites possibly at Ser35, Ile42, or Lys55, while consistently terminating at Asn441 at the C-terminus. Furthermore, Lp-PLA2 possesses two potential N-linked glycosylation sites at Asn423 and Asn433 (4). The gene encoding this enzyme is situated on human chromosome 6p12–p21.1, consisting of 12 exons, and encodes a secreted phospholipase known as Lp-PLA2, also referred to as PAF-AH. This protein is a member of the Ca²^+^-independent phospholipase A2 family, with a molecular weight of approximately 45 kDa (4). The protein comprises 441 amino acids and exhibits a characteristic α/β hydrolase folding and it is characterized by a central octameric mixed β-sheet flanked by α-helices. Its primary structure retains the classic Gly-X-Ser-X-Gly lipase motif (18). Furthermore, the protein structure includes two hydrophobic helices, located approximately at residues 114–126 and 360–368, which are hypothesized to facilitate its binding to phospholipid membranes, such as LDL surfaces and HDL (19), thereby forming the interface for lipid interaction. Unlike most members of the phospholipase A2 family that require calcium for catalytic activity, Lp-PLA2 is a calcium-independent secreted hydrolase that specifically acts on oxidized or short-chain phospholipids. Its primary substrates include PAF and Lp-PLA2 oxidized phospholipids present in oxLDL. During the hydrolysis of PAF, Lp-PLA2 converts it into inactive LysoPAF, thereby exerting anti-inflammatory and immunosuppressive effects. In contrast, the degradation of oxidized phospholipids results in the formation of LysoPC and free fatty acids, metabolites that strongly induce inflammatory responses. Consequently, Lp-PLA2 exhibits a classic “double-edged sword” effect in immune-inflammatory processes. The enzyme establishes an amphipathic binding interface surrounding its catalytic pocket, characterized by a high concentration of hydrophobic and aromatic residues, which enhances the recognition and binding of a variety of phospholipid substrates. Structural analyses and hydrogen-deuterium exchange mass spectrometry (HDX-MS) studies reveal that critical hydrophobic residues, such as Trp97, Leu107, Phe110, Leu121, Phe125, Phe156, Phe357, Ile365, and Leu369, contribute to the stabilization of substrates with oxidized or short-chain fatty acid groups through π-π stacking and hydrophobic interactions (20). Additionally, the surface of Lp-PLA2 displays distinct amphiphilic properties: one side presents hydrophilic residues for interaction with phospholipid head groups, while the opposite side facilitates binding to the lipid environment through hydrophobic residues, thereby promoting the transport of substrates from the lipoprotein membrane into the catalytic center (4, 17).

#### 2.1.2 Substrate specificity

Lp-PLA2 demonstrates a broad substrate spectrum, efficiently cleaving phospholipids with short-chain or oxidized groups beyond the hydrolysis of PAF. Notably, it exhibits optimal activity toward phosphatidylcholine (PC) with sn-2 carbon chain lengths of nine or fewer, targeting various short-chain acyls and oxidized PC substrates that contain carboxyl or aldehyde groups (19, 20). *In vitro* studies on substrate specificity indicate that PAF exhibits the highest affinity for Lp-PLA2, followed by oxidized acyl derivatives such as butyryl, butanoic acid, and 5′-oxovaleroyl. Further investigations have revealed that Lp-PLA2 also cleaves oxidized phospholipids located on atherosclerotic plaques and apoptotic cell surfaces, including oxidized phosphatidylserine (PSox) and oxidized phosphatidylethanolamine (oxPE) (21), The substrate range of Lp-PLA2 is notably broad. Predominantly, Lp-PLA2 targets phosphatidylcholine substrates characterized by short acyl chains or oxidative modifications, resulting in the production of lysophosphatidylcholine (LPC) and oxidized fatty acids. The enzyme’s interaction with the lipid monolayer is a pivotal phase in its catalytic mechanism, facilitating conformational alterations necessary for substrate extraction (22).

**Figure 3:**
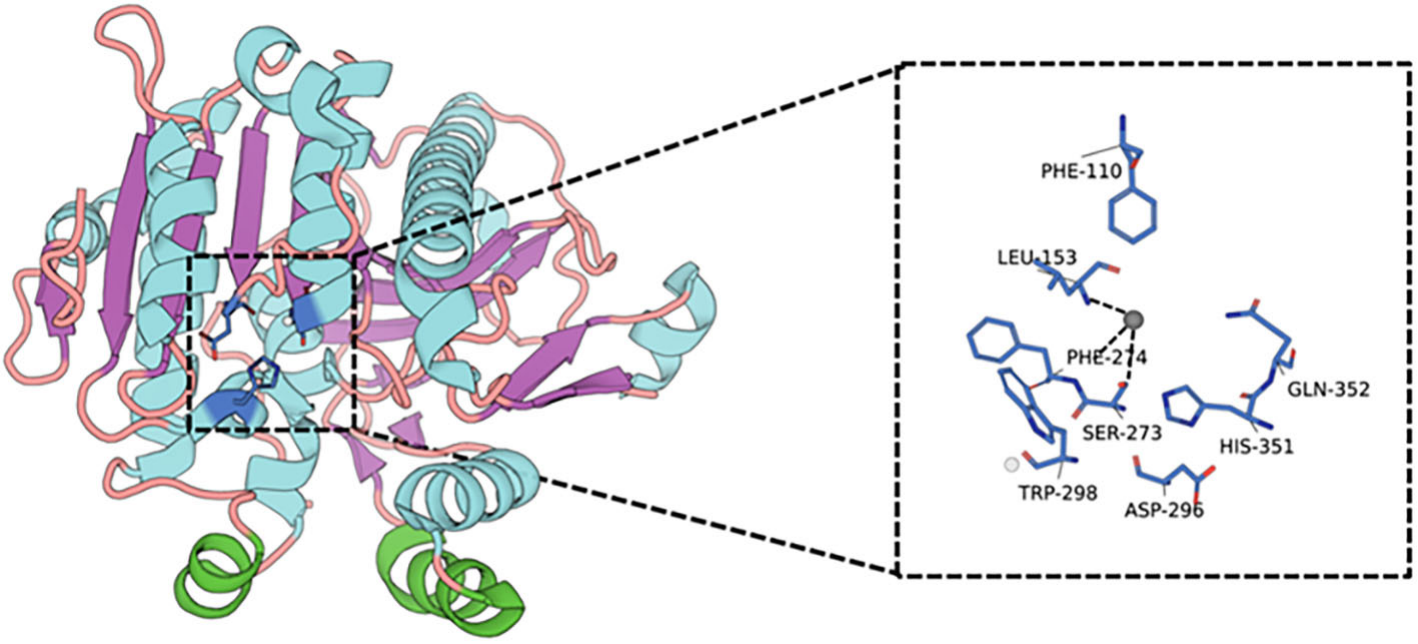
Structural representation of human Lp-PLA2 based on the crystal structure (PDB: 3D59). The three-dimensional architecture of Lp-PLA2 is visualized using PyMOL and rendered with a secondary-structure–based coloring scheme, in which α-helices, β-strands, and loops are distinguished by different colors. Lp-PLA2 adopts a canonical α/β-hydrolase fold. The dashed box indicates the catalytic pocket, where key residues essential for substrate binding and catalysis are shown in stick representation, including the catalytic triad (Ser273, Asp296, His351) and surrounding hydrophobic or aromatic residues (Phe110, Leu153, Phe214, Trp298, Gln352) that shape the substrate-entry channel. This structural configuration enables Lp-PLA2 to extract oxidized phospholipids from lipoprotein surfaces and correctly position them into the active site for hydrolysis.

Structural investigations have identified a classic αβ hydrolase fold, with a catalytic triad consisting of Ser273, Asp296, and His351 (22–24). The polar headgroup of the substrate engages with specific residues through hydrogen bonding: the phosphate moiety interacts with Arg218, the carbonyl oxygen of the acyl glycerol backbone forms a hydrogen bond with Gln211, and the choline’s positive charge aligns with a negatively charged region on the enzyme’s surface (19). The sn-2 acyl binding site is characterized as a relatively enclosed hydrophobic pocket, constituted by small hydrophobic residues such as Ala155, Leu159, and Ala355, in conjunction with residues like His151, Tyr160, and His272 (19). Short acyl chains comprising 2 to 4 carbon atoms can be fully accommodated within this pocket, whereas longer terminal chains with aldehyde or carboxyl groups exhibit increased affinity due to the formation of additional hydrogen bonds with residues such as His272 and Tyr160. Notably, Lp-PLA2 is characterized by the absence of a conventional “lid” structure, resulting in its active site being directly exposed to the lipid-water interface, which facilitates direct substrate extraction from plasma lipoprotein monolayers (22, 25). Additionally, the enzyme’s surface features a hydrophobic α-helical region, approximately spanning positions 113 to 120, which includes hydrophobic residues such as Trp115, Leu116, and Tyr205. This region can integrate into the lipid monolayer, thereby stabilizing the enzyme’s positioning on the lipoprotein surface (25–27). These structural characteristics, encompassing the hydrophobic channel surrounding the active site and the spatial arrangement of polar residues, are critical in determining substrate specificity. To consolidate the mechanistic details described above, the key catalytic residues, structural domains, and lipid-binding features of Lp-PLA2 are summarized in **Table 1**. Furthermore, the spatial arrangement of the catalytic triad and the substrate-entry channel is shown in **Figure 3**, which provides a structural basis for the enzyme’s selectivity toward oxidized sn-2 acyl chains.

**Table 1:** Key catalytic residues, structural domains, and hydrolytic products of Lp-PLA2.

Category	Key elements	Description/Functional role	References
Catalytic Residues	Ser273	Nucleophilic residue of the catalytic triad; initiates hydrolysis by attacking the sn-2 ester bond of phospholipids.	(22–24)
	Asp296	Stabilizes His351 through hydrogen-bonding interactions; maintains catalytic geometry.	(22–24)
	His351	Acts as a general acid/base; abstracts a proton from Ser273 and facilitates substrate cleavage.	(22–24)
Structural/Binding Domains	Gly-X-Ser-X-Gly lipase motif	Classical lipase consensus sequence containing the catalytic serine; characteristic of α/β-hydrolases.	(18)
	N-terminal regulatory segment (Met1–Ala17; alternative starts Ser35/Ile42/Lys55)	Functions as a secretory signal peptide and contributes to N-terminal heterogeneity.	(4, 17)
	N-linked glycosylation sites (Asn423, Asn433)	May influence folding, stability, and secretion of Lp-PLA2.	(4)
	Hydrophobic helices (~114–126; 360–368)	Facilitate interaction with phospholipid monolayers of LDL and HDL, forming the membrane-binding interface.	(19)
	Hydrophobic substrate-binding pocket	Composed of residues such as Trp97, Leu107, Phe110, Leu121, Phe125, Phe156, Phe357, Ile365, Leu369; stabilizes oxidized or short-chain acyl groups.	(19, 20)
	Polar head-group recognition region	Arg218 and Gln211 form hydrogen bonds with the phosphate and acyl glycerol backbone of substrates.	(19)
	sn-2 acyl binding pocket	Formed by Ala155, Leu159, Ala355, His151, Tyr160, His272; accommodates short-chain or oxidatively modified sn-2 acyl groups.	(19)
Hydrolytic Products	LysoPAF	Inactive product of PAF hydrolysis; contributes to anti-inflammatory effects.	(2, 18)
	LPC	Strongly pro-inflammatory product of oxidized PC hydrolysis; promotes atherosclerosis.	(19, 26)
	Oxidized free fatty acids (oxFFA)	Generated from oxidized phospholipids; stimulate inflammation and plaque instability.	(19, 26)
	LPE	Produced during PE hydrolysis; Lp-PLA2 inhibition reduces LPE and increases intracellular PE in tumor cells.	(21)
	Deacetylated PAF analogs	Hydrolysis products of PAF-like substrates; involved in immunomodulation.	(2, 18)

In comparison to other members of the PLA2 family, Lp-PLA2 demonstrates distinctive substrate specificity. For instance, cytoplasmic PLA2(cPLA2, Group IV) is a high-molecular-weight, Ca²^+^-dependent protein that associates with membranes via its C2 domain and predominantly targets PC/PE/PI substrates containing arachidonic acid (AA, 20:4) (28, 29); Secretory phospholipase A2(SPLA2) enzymes, specifically those belonging to groups I, II, and X, are characterized by their low molecular weight and dependence on calcium ions (Ca²^+^) for activity. These enzymes exhibit distinct substrate preferences across different subtypes: the IIA group predominantly targets phosphatidylcholine (PC) and phosphatidylethanolamine (PE), whereas groups V and X show a preference for unsaturated long-chain acyl groups (25, 30). In contrast, the calcium-independent phospholipase A2 (iPLA2) of group VI is a macromolecular enzyme with broad substrate selectivity, playing a significant role in the remodeling of membrane phospholipids. On the other hand, Lp-PLA2 operates independently of calcium ions, with its open active site specifically accommodating short-chain or oxidized acyl groups. This mechanism is fundamentally distinct from the calcium-dependent insertion and activation observed in other PLA2 isoforms (25).

Lp-PLA2 demonstrates a high degree of specificity in substrate recognition, with particular sensitivity to the nature of the sn-2 residue. An increase in chain length at this position significantly diminishes its hydrolytic efficiency. Additionally, Lp-PLA2 can cleave short-chain diacylglycerols, triacylglycerols, and acetylated alkanols, suggesting a lack of strict dependence on polar headgroups. The enzyme’s recognition of the sn-1 position is relatively flexible, as it can act on residues linked by either ester or ether bonds. Consequently, any glyceride possessing a hydrophobic group at the sn-1 position and an ester bond at sn-2 may serve as a substrate. Moreover, under specific conditions, Lp-PLA2 exhibits phospholipase A_1_ activity, enabling the cleavage of the sn-1 acyl group (1, 18). Importantly, truncated phospholipids with shortened or oxidized sn-2 chains are primarily produced through oxidative stress affecting the phospholipid components of membranes and lipoproteins (19).

In pathological conditions such as atherosclerosis, inflammation, and tumorigenesis, the substrate utilization of Lp-PLA2 becomes increasingly reliant on variations in accessible substrate types, although its substrate preference characteristics remain unchanged. For instance, in atherosclerotic plaques enriched with oxLDL, Lp-PLA2 predominantly cleaves oxidized phosphatidylcholine (PC), resulting in the production of LPC and oxidized free fatty acids, which in turn promote inflammation and enhance plaque instability (26). Under conditions of apoptotic or oxidative stress, phosphatidylserine oxidation (PSox) emerges as a novel substrate for Lp-PLA2, to which it exhibits high specificity. The hydrolysis of PSox by Lp-PLA2 impairs the clearance of apoptotic cells by macrophages. Furthermore, studies related to tumors have demonstrated that Lp-PLA2 plays a regulatory role in phospholipid metabolism within cancer cells. The deletion or inhibition of Lp-PLA2 results in increased intracellular levels of phosphatidylethanolamine (PE) and decreased levels of lysophosphatidylethanolamine (LPE), indicating that Lp-PLA2 typically cleaves PE (21). In conclusion, while the types of substrates encountered by Lp-PLA2 vary across different pathological states, its preference for cleaving short-chain or oxidatively modified phospholipids remains consistent, as corroborated by experimental evidence.

#### 2.1.3 Structural-function relationships revealed by mutation studies

Investigations into the polymorphisms of Lp-PLA2 have provided deeper insights into the functional importance of its structural characteristics. The extensively studied V279F mutation results in abnormal protein folding, leading to almost undetectable levels of active protein in plasma, which suggests impaired secretion (4). Structural analyses indicate that the residues V279 and the adjacent Gln281 are located near the catalytic pocket entrance; mutations at these positions are likely to disrupt the conformation of the active site. Another common variant, I198T (also known as I198Thr), does not affect the maximum reaction rate (Vmax) but increases the Km value by approximately sixfold, indicating a significant decrease in substrate affinity and emphasizing the crucial role of residue 198 in substrate binding. Furthermore, the R92H and V379A mutations, which are situated on the protein surface, are linked to diminished lipoprotein-binding capacity, possibly due to alterations in hydrophobic interfaces that compromise the interaction between Lp-PLA2 and LDL/HDL (4). Taken together, these mutational analyses underscore the vital contributions of specific residues to enzymatic activity and substrate recognition.

### 2.2 Biological functions and disease impacts of Lp-PLA2

Lp-PLA2 encoded by PLA2G7, has emerged as an important enzyme linking lipid metabolism with inflammation. Although early studies proposed that Lp-PLA2 may exert anti-inflammatory effects by degrading PAF, accumulating evidence indicates that the LysoPC and oxNEFA produced by its enzymatic activity act as potent pro-inflammatory mediators (18, 31–33).

The biological impact of Lp-PLA2 strongly depends on the lipoprotein particles to which it binds. When associated with LDL or lipoprotein(a), Lp-PLA2 promotes endothelial activation, oxidative stress, and inflammatory signaling. In contrast, Lp-PLA2 bound to HDL has been reported to exhibit potential anti-inflammatory effects (1, 34). However, the physiological significance of these differences remains incompletely understood, as most measurable plasma Lp-PLA2 activity reflects the enzyme bound to LDL. These functional disparities suggest that the lipoprotein context modulates Lp-PLA2–mediated lipid metabolism and inflammatory responses.

In the circulation, Lp-PLA2 is a 45−kDa secreted phospholipase (also called plasma PAF-acetylhydrolase) made primarily by macrophages (35). It is not free in plasma but almost entirely bound to lipoproteins – roughly two-thirds of plasma Lp-PLA2 associates with LDL particles and the rest with HDL. Lp-PLA2 specifically hydrolyzes oxidized phospholipids (especially oxidized phosphatidylcholine) in LDL, cleaving the sn−2 acyl bond. This reaction liberates LysoPC and oxNEFA (35, 36). Because LysoPC and oxNEFA are small, water−soluble molecules, they diffuse into the plasma aqueous phase rather than staying attached to the lipoprotein particle. These soluble products (LysoPC and oxNEFAs) are bioactive and contribute to vascular inflammation and endothelial activation (36).

**Figure 4:**
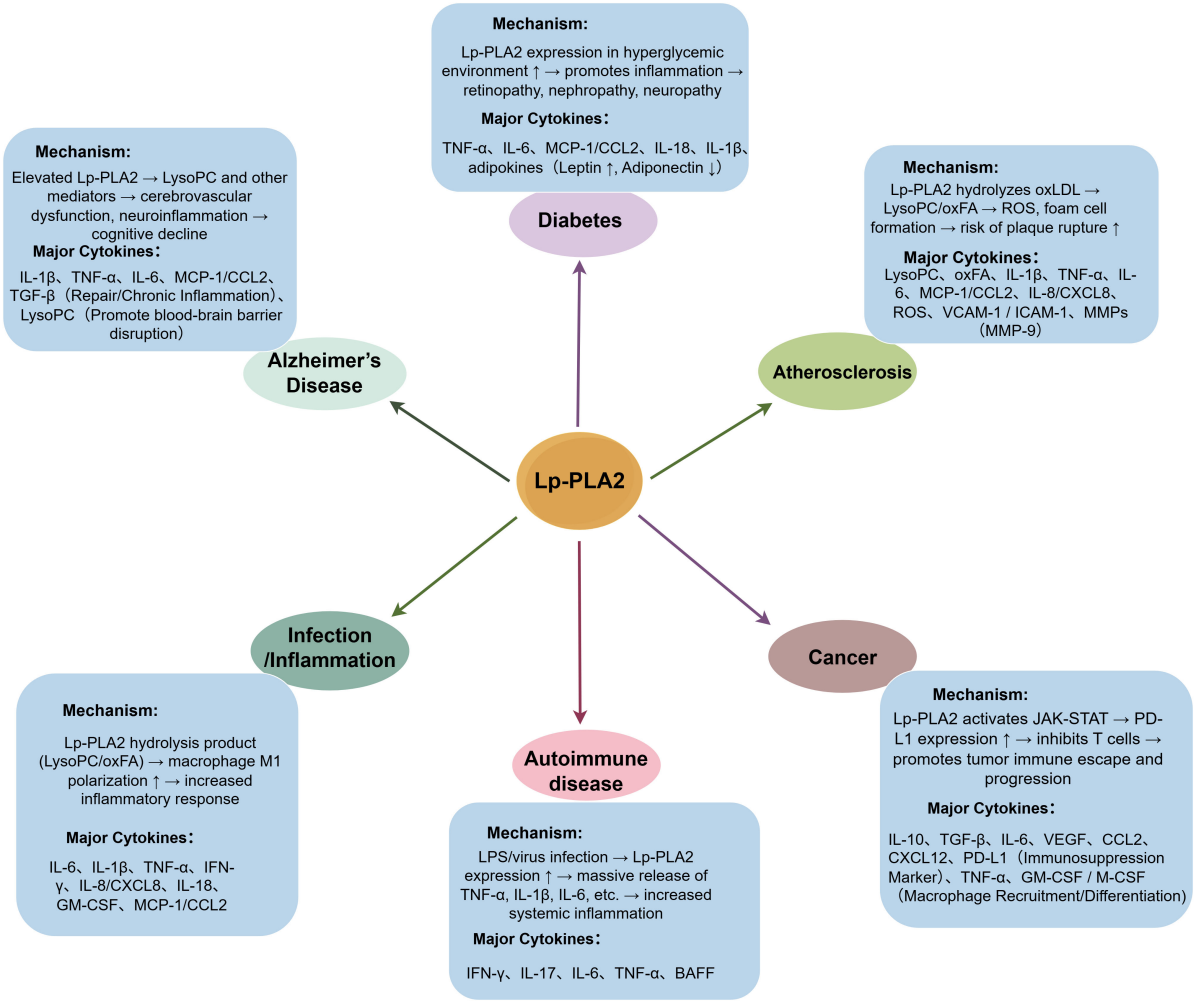
Diagram illustrating Lp-PLA2’s role in various diseases with colored boxes and arrows. Central orange circle labeled “Lp-PLA2” connects to conditions: Alzheimer's Disease, Diabetes, Atherosclerosis, Cancer, Infection/Inflammation, Autoimmune Disease. Each condition has a blue box detailing mechanisms and major cytokines involved, affecting processes like inflammation, cognitive decline, and tumor progression.

Recent studies using animal models, clinical cohorts, and genetic analyses have expanded our understanding of Lp-PLA2 across several major disease categories, including cardiovascular-metabolic disorders, neurodegenerative diseases, cancers, and autoimmune conditions (37). A panoramic overview of Lp-PLA2-associated diseases is presented in **Figure 4**. Rather than acting through a single mechanism, Lp-PLA2 participates in disease progression through multiple pathways involving lipid oxidation, macrophage activation, endothelial dysfunction, and immune regulation.

Lp-PLA2 influences disease pathogenesis by worsening inflammation in cardiovascular and metabolic disorders, affecting neuroinflammation in Alzheimer’s, aiding immune evasion in tumors, and altering immune responses in autoimmune diseases. This complexity underscores the need for careful interpretation of Lp-PLA2 activity across different pathological environments and highlights the importance of targeted therapeutic strategies, which are further discussed in Section 4.

#### 2.2.1 Atherosclerosis

The onset and advancement of atherosclerotic vascular disease are often linked to the generation of bioactive lipid mediators and the elicitation of vascular inflammatory responses. This represents a systemic pathological process involving the collaborative participation of inflammatory and immune mechanisms. Lp-PLA2, an enzyme primarily secreted by inflammatory cells such as macrophages, is predominantly localized within the necrotic core and fibrotic cap of vulnerable plaques. Lp-PLA2 binds to LDL and hydrolyzes oxidized phospholipids within it. Moreover, mast cells, platelets, monocytes, dendritic cells, and the liver are also capable of producing Lp-PLA2 (38). This enzyme contributes to endothelial dysfunction, exacerbates plaque inflammation, and facilitates the formation of necrotic cores within atherosclerotic plaques by hydrolyzing modified and oxidized phospholipids on the surface of LDL cholesterol (LDL-C) and within arterial plaques (39). The enzyme in question releases products such as arachidonic acid, LysoPC, and oxidized fatty acids, which are well-established as critical initiators of inflammatory responses. Research indicates that Lp-PLA2 is intricately linked to inflammation within atherosclerotic plaques, with its hydrolytic product, LysoPC, playing a crucial role in exacerbating plaque inflammation and increasing vulnerability (40). The accumulation of LysoPC and oxidized fatty acids in the subendothelial layer contributes to the development of the lipid “core” within atherosclerotic plaques. Macrophages that internalize these substrates undergo a markedly accelerated transformation into foam cells. Simultaneously, LysoPC activates endothelial nicotinamide adenine dinucleotide phosphate hydrogen(NADPH) oxidase and induces the “uncoupling” of endothelial nitric oxide synthase (eNOS), resulting in the substantial production of reactive oxygen species (ROS), including superoxide radicals (42). This process further facilitates enzymatic reactions, rendering superoxide and peroxynitrite significant contributors to oxidative stress. Consequently, this accelerates the progression of atherosclerosis and heightens plaque instability.

**Figure 5:**
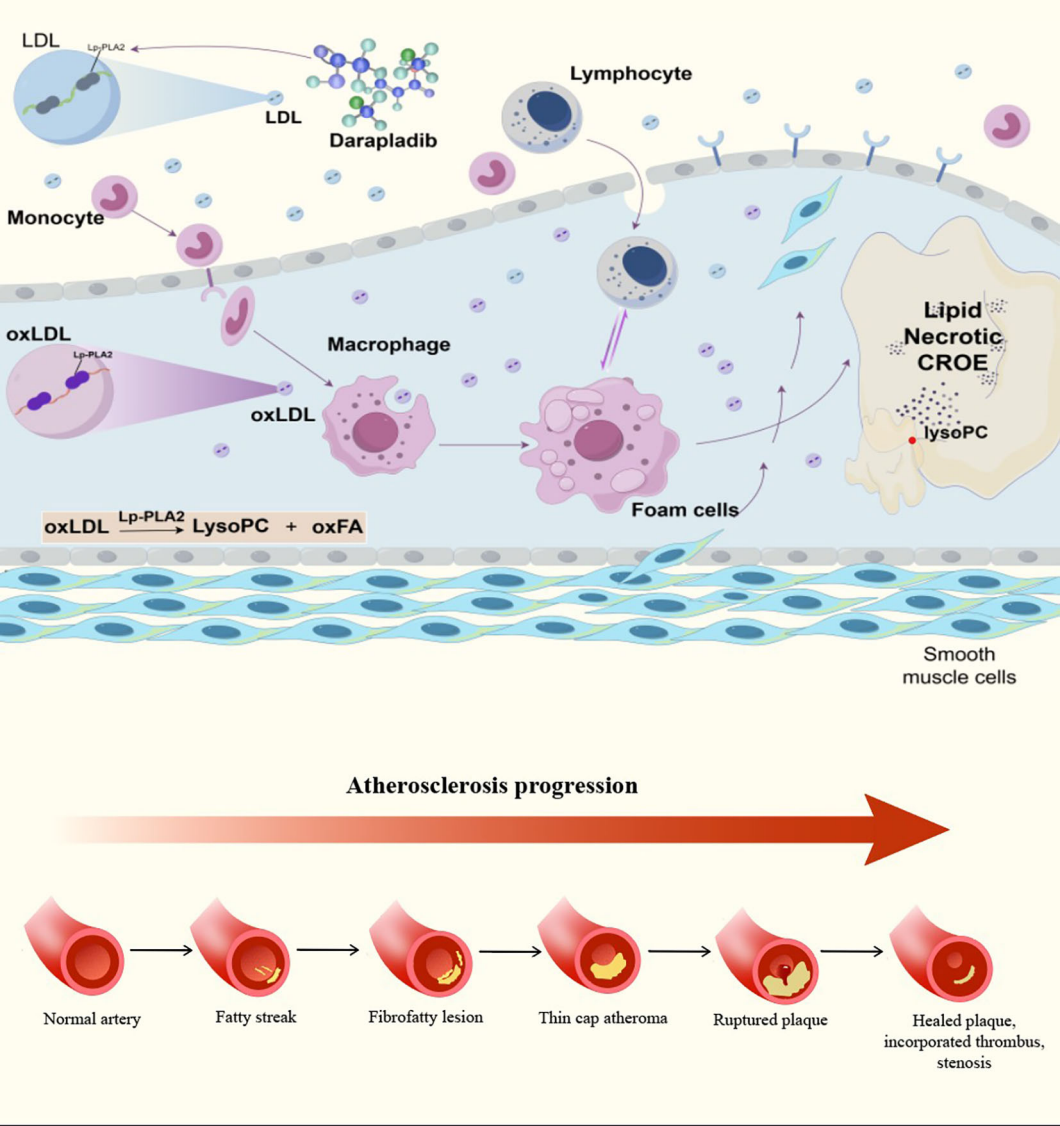
Mechanistic role of Lp-PLA2 in atherosclerotic plaque development and the life-cycle of plaque progression. The upper panel illustrates the involvement of Lp-PLA2 in the formation and progression of atherosclerotic plaques. LDL enters the subendothelial space and becomes oxidized to form oxLDL, which promotes monocyte adhesion and recruitment. Macrophages internalize oxLDL and differentiate into foam cells, contributing to lipid accumulation and the development of a necrotic core. Lp-PLA2 hydrolyzes oxLDL to generate LysoPC and oxFA, which amplify endothelial dysfunction, inflammatory activation, and plaque instability. Darapladib inhibits Lp-PLA2 activity and reduces pro-inflammatory lipid mediator generation. The lower panel depicts the sequential stages of the atherosclerotic plaque life-cycle: normal artery → fatty streak → fibrofatty lesion → thin-cap atheroma → ruptured plaque → healed plaque with stenosis, consistent with the classical rupture-prone progression model. Adapted from Libby et al., 2021 and Bentzon et al., 2014.

In the initial phases of acute coronary syndrome (ACS), there are notable dynamic alterations in serum levels of Lp-PLA2. Numerous clinical studies have demonstrated that elevated Lp-PLA2 serves as an independent predictor of cardiovascular event outcomes in ACS patients, even after controlling for potential confounding variables. Among the elderly Chinese population, higher concentrations of Lp-PLA2 are significantly associated with increased risks of carotid atherosclerosis, myocardial infarction, and cardiovascular mortality (42). Experimental research suggests that Lp-PLA2 plays a role in the multistage progression of atherosclerotic lesions through its proinflammatory and prooxidative effects. The stepwise involvement of Lp-PLA2 in lipoprotein oxidation, endothelial activation, macrophage recruitment, and plaque maturation is illustrated in **Figure 5**, which integrates the enzyme into the life-cycle of atherosclerotic plaque progression. In addition to traditional cardiovascular risk factors, Lp-PLA2 shows promise as a biomarker for predicting clinical outcomes in patients with coronary heart disease. Elevated levels of Lp-PLA2 are associated with adverse outcomes in patients with coronary artery disease (CAD), indicating its potential involvement in disease progression through compromised endothelial function and the structural stability of arterial walls (43). In atherosclerotic cardiovascular disease (ASCVD), Lp-PLA2 is identified as an independent risk biomarker that facilitates vascular inflammation by modulating lipid metabolism in the bloodstream and plaques (44). Additional evidence suggests that Lp-PLA2 functions not only as a risk factor for atherosclerosis but also as an independent predictor of plaque vulnerability (45). During the progression of atherosclerosis, Lp-PLA2 contributes to plaque instability and the risk of rupture through pro-inflammatory mechanisms, thereby elevating the incidence of cardiovascular events (46). Notably, Lp-PLA2 levels in carotid plaques have been significantly correlated with plaque vulnerability, highlighting its pivotal role in the pathogenesis of atherosclerosis. Moreover, Lp-PLA2 acts in concert with other inflammatory markers, such as C-reactive protein (CRP), to enhance the inflammatory response associated with atherosclerosis (46). Certain interventions, including α-lipoic acid supplementation, have been suggested to mitigate cardiovascular disease risk by modulating Lp-PLA2 activity and its distribution within lipoproteins (47).

As a potential therapeutic target, the development of Lp-PLA2 inhibitors has garnered significant attention, although the oral inhibitor darapladib showed promise in preclinical studies, it failed to significantly reduce cardiovascular event risk in two pivotal Phase III trials (STABILITY and SOLID-TIMI 52) (48, 49). The failure of clinical trials may be attributed to several factors, including the widespread use of statins, the lack of stratification by inflammatory markers such as high-sensitivity C-reactive protein (hsCRP), and the short duration of follow-up. Additionally, pharmacogenetic analyses have not identified significant associations between variants related to Lp-PLA2 activity and cardiovascular endpoints (4). These findings also suggest that lysophosphatidylcholine can be generated non-enzymatically through the spontaneous deacylation of oxidized phospholipids. This process is unaffected by Lp-PLA2 inhibitors and may partially explain the negative outcomes observed in clinical trials. Despite these challenges, Lp-PLA2 is widely recognized as a reliable biomarker for assessing cardiovascular risk. Its levels show a significant correlation with cardiovascular events and maintain their independence after multivariate adjustment (50). Furthermore, Lp-PLA2 levels are closely associated with the severity of carotid stenosis and the incidence of stroke events in high-risk populations (50). Research suggests that Lp-PLA2 plays a crucial role in plaque formation and instability by releasing proinflammatory mediators through the hydrolysis of oxLDL (51). Although clinical trials targeting Lp-PLA2 inhibition have produced inconsistent results concerning coronary events, Lp-PLA2 remains a focal point of research in the inflammatory processes underlying atherosclerosis (52, 53).

Lp-PLA2 demonstrates varying predictive value across different cardiovascular diseases. It is associated with an increased risk of heart failure following acute myocardial infarction and is significantly linked to disease progression and plaque stability in atherosclerotic cardiovascular disease (ASCVD) (44). These observations indicate that Lp-PLA2 not only assists in forecasting cardiovascular events but also holds potential for enhancing personalized medicine and risk stratification (54). In the context of metabolic diseases, Lp-PLA2 plays a crucial role: its expression is elevated in individuals with obesity and type 2 diabetes, correlates with adverse lipid profiles, and may contribute to dyslipidemia by modulating LDL-C and oxLDL metabolism (55). I In non-alcoholic steatohepatitis (NASH), increased Lp-PLA2 expression promotes autophagy by inhibiting the JAK2/STAT3 pathway, thereby mitigating the progression of NASH (56). In oncology, Lp-PLA2 may have protective effects; for example, reduced serum Lp-PLA2 levels in lung cancer patients with pleural effusion suggest its potential as an adjunctive diagnostic and staging marker (52). Furthermore, elevated levels of Lp-PLA2 in patients with COPD are associated with the severity of the disease and exercise tolerance, thereby underscoring its potential utility as a biomarker for COPD (57). Overall, Lp-PLA2 exerts a complex and pivotal regulatory influence across various pathological conditions—including cardiovascular disease, metabolic disorders, cancer, and chronic inflammation—attributable to its role in modulating lipid metabolism and its origins from multiple cell types. This establishes Lp-PLA2 as a target of considerable research interest and clinical potential. These disease-specific mechanisms align with the integrated pathological framework summarized in **Figure 4**, highlighting shared cytokine-driven inflammatory circuits across multiple disorders.

#### 2.2.2 Asthma and allergies

Lp-PLA2, predominantly secreted by macrophages, catalyzes the hydrolysis of phospholipids in oxidized low-density lipoproteins, resulting in the production of pro-inflammatory mediators such as LysoPC. Concurrently, this enzyme plays a role in modulating inflammatory responses as a PAF. Genetic research suggests that polymorphisms in the Lp-PLA2 gene are linked to an increased susceptibility to atopic asthma. Clinically, elevated levels of PAF have been significantly detected in the airway secretions of asthma patients, whereas Lp-PLA2 is known to effectively degrade PAF (58–60), suggesting potential interactions between these molecules in allergic inflammation. Lp-PLA2 activity negatively correlates with asthma severity, decreasing as disease worsens (61), Despite the fact that only 20–30% of Lp-PLA2 associates with HDL (58). asthma patients exhibit elevated levels of PAF—the substrate for Lp-PLA2—in both sputum and bronchoalveolar lavage fluid compared to non-asthmatic individuals (59). However, a study reported no significant difference in serum Lp-PLA2 levels between individuals with allergic asthma and healthy controls, indicating limited specificity and sensitivity of Lp-PLA2 as a diagnostic biomarker for asthma. To date, no Lp-PLA2 inhibitors have been developed for the treatment of asthma. In the realm of cardiovascular disease research, clinical trials involving the inhibitor darapladib have similarly failed to demonstrate the expected efficacy. Therefore, the precise role of Lp-PLA2 in asthma and allergic reactions remains ambiguous, and further research is necessary to evaluate its potential as a biomarker or therapeutic target. These disease-specific mechanisms align with the integrated pathological framework summarized in **Figure 4**, highlighting shared cytokine-driven inflammatory circuits across multiple disorders.

#### 2.2.3 Alzheimer’s disease

The onset and progression of Alzheimer’s disease (AD) are characterized by significant vascular pathological changes. The enzyme Lp-PLA2 catalyzes the oxidation of phospholipids, resulting in the generation of bioactive mediators, such as LysoPC, which subsequently impair vascular endothelial function and compromise the integrity of the blood-brain barrier.

Empirical evidence indicates that plasma levels of Lp-PLA2 are significantly elevated in individuals with AD. Those with Lp-PLA2 levels above the median exhibit approximately a 1.9-fold increased risk of developing AD compared to the general population (62), suggesting that Lp-PLA2 may contribute to AD pathogenesis through mechanisms involving vascular injury. Proteomic analyses further demonstrate a marked upregulation of Lp-PLA2 in the plasma of patients with mild cognitive impairment (MCI) and AD. Diagnostic models that integrate multiple biomarkers, including Lp-PLA2, effectively differentiate between healthy controls, MCI, and AD patients. Notably, Lp-PLA2 exhibited an area under the receiver operating characteristic (ROC) curve of 0.9744 in distinguishing AD from controls (63, 64), underscoring its strong diagnostic potential.

Longitudinal studies have further validated the prognostic value of Lp-PLA2 in predicting cognitive decline. In a large prospective cohort study, Fitzpatrick et al. reported hazard ratios (HR) of 2.21 (95% CI: 1.12–4.37) for all-cause dementia and 1.98 (95% CI: 1.22–3.21) for AD when comparing the highest versus lowest quartiles of Lp-PLA2 mas (62). These findings suggest that Lp-PLA2 not only serves as a diagnostic marker but also as a predictor of disease progression, potentially identifying individuals at high risk for rapid cognitive deterioration.

Despite its low expression in the central nervous system, Lp-PLA2’s systemic pro-inflammatory effects have been associated with cognitive decline. Given the critical role of vascular and neuroinflammation in AD, Lp-PLA2 has emerged as a potential therapeutic target (65, 66). Preclinical studies and early clinical trials indicate that Lp-PLA2 inhibitors, such as darapladib, may enhance blood-brain barrier (BBB) function and mitigate cognitive deterioration (67). In a Phase II clinical trial, darapladib treatment for 24 weeks in mild-to-moderate AD patients demonstrated trends toward improved cognitive function and reduced cerebrospinal fluid (CSF) inflammatory markers, although the primary efficacy endpoint was not met (67). These results suggest potential benefit in specific AD subpopulations, particularly those with elevated vascular inflammation markers. Consequently, Lp-PLA2 is currently recognized as a pro-inflammatory factor implicated in the progression of AD (62, 68), although its specific mechanisms and clinical utility require further validation.

Mechanistically, LysoPC generated through Lp-PLA2 hydrolysis exerts pathological actions relevant to neuroinflammation. LysoPC (LPC) has been shown to activate innate immune signaling and to induce NOD-like receptor family pyrin domain containing 3(NLRP3) inflammasome–dependent responses in immune and endothelial cells; LPC-triggered NLRP3 activation leads to caspase-1 cleavage and release of mature interleukin-1 beta (IL-1β)/interleukin-18 (IL-18), thereby amplifying local inflammatory cascades that are deleterious for neurons and synapses (69). In parallel, LPC and other oxidized lipid species induce inflammatory stress in brain microvascular endothelial cells (BMECs), disrupt tight junction proteins and increase paracellular permeability, thereby impairing BBB function and facilitating peripheral immune cell entry into the parenchyma. Such lipid-driven endothelial dysfunction provides a mechanistic link between systemic vascular inflammation and central neuroinflammation (70).

Recent studies have further elucidated the role of Lp-PLA2 in Aβ metabolism and tau pathology. LysoPC-induced endothelial dysfunction impairs Aβ clearance across the BBB through reduced expression of low-density lipoprotein receptor-related protein 1 (LRP1), a major Aβ efflux transporter (71). Furthermore, chronic exposure to LysoPC in neuronal cultures promotes tau hyperphosphorylation through activation of glycogen synthase kinase-3β (GSK-3β) and cyclin-dependent kinase 5 (CDK5), key kinases implicated in tau pathology (72). These findings suggest that Lp-PLA2-mediated lipid products directly contribute to the core pathological hallmarks of AD beyond their vascular effects.

The core neuropathological features of AD include β-amyloid (Aβ) deposition and neurofibrillary tangles formed by hyperphosphorylated tau protein, which represent the primary cause of dementia in the elderly. Accumulating evidence indicates that chronic neuroinflammation and BBB disruption constitute central pathological mechanisms that drive AD progression (71). In the AD brain, persistently activated microglia secrete pro-inflammatory factors such as tumor necrosis factor Alpha (TNF-α) and IL-1β, which exacerbate Aβ and tau pathology and promote neurotoxicity (72). These cytokines can destabilize endothelial tight junctions (occludin, claudin-5, ZO-1) and increase BBB permeability, facilitating peripheral immune cell infiltration and establishing a self-perpetuating cycle of neuroinflammation and neuronal damage (71).

The interaction between Lp-PLA2 and apolipoprotein E (APOE) genotype may further modulate AD risk. In APOE ϵ4 carriers, LDL-bound Lp-PLA2 activity is significantly higher than in non-carriers, potentially due to altered lipoprotein composition and enhanced oxidative modification (73). This synergistic effect between Lp-PLA2 and APOE ϵ4 may explain the accelerated cognitive decline observed in individuals carrying both high Lp-PLA2 levels and the APOE ϵ4 allele (73).

The BBB, a highly specialized brain endothelial structure and a critical component of the neurovascular unit, exhibits dysfunction that is closely linked to AD pathogenesis. Through coordinated interactions with astrocytes and microglia, the BBB meticulously regulates the exchange of substances between the bloodstream and brain tissue, thereby maintaining the homeostasis of the central nervous system’s internal environment (74). The tight junctions between cerebral vascular endothelial cells, primarily composed of occludin, claudin, and junctional adhesion molecules, are essential for preserving the structural and functional integrity of the BBB. Disruption of these tight junctions by inflammation or other pathological factors can increase BBB permeability, allowing harmful substances to infiltrate brain tissue and potentially accelerate the progression of AD (75). Recent research by Nation et al. has demonstrated that cerebral capillary damage and BBB disruption are already evident in the hippocampal region of patients with early cognitive impairment. They suggest that compromised BBB integrity could serve as an early biomarker for cognitive impairment, independent of amyloid-beta (Aβ) or phosphorylated tau (pTau) pathology (76). This finding implies that neurovascular dysfunction may be an underestimated contributor to the pathogenesis of AD. In this context, Lp-PLA2 recognized as a marker of vascular inflammation, has attracted considerable attention for its potential role in AD (71).

Nevertheless, the literature presents mixed findings regarding the association between Lp-PLA2 and AD. For instance, Davidson et al. reported no significant correlation between plasma Lp-PLA2 activity and AD diagnosis, nor did they find any clear associations with AD-related CSF biomarkers (77, 78). In contrast, a case-control study indicated that elevated plasma Lp-PLA2 levels are independently associated with an increased risk of AD and may synergistically augment the incidence of AD in conjunction with cardiovascular disease (62, 73). Furthermore, in a study on dementia, Fitzpatrick et al. identified hazard ratios (HR) of 2.21 (95% CI: 1.12–4.37) and 1.98 (95% CI: 1.22–3.21) for the highest versus lowest Lp-PLA2 mass quartiles, thereby reinforcing the notion of Lp-PLA2 as a risk factor for AD (62). Based on well-designed, adequately powered, and clearly defined studies, we infer a potential association between Lp-PLA2 and AD risk. Overall, despite some inconsistencies in the evidence, Lp-PLA2 may constitute an independent risk factor for AD. These disease-specific mechanisms align with the integrated pathological framework summarized in **Figure 4**, highlighting shared cytokine-driven inflammatory circuits across multiple disorders.

#### 2.2.4 Diabetes

In the context of diabetic macrovascular disease, significant changes in Lp-PLA2 levels have been documented, showing a strong correlation with fasting blood glucose levels and cardiovascular outcomes in patients with diabetes (4). Elevated expression of Lp-PLA2 is consistently observed in individuals with type 2 diabetes mellitus (T2DM) and obesity. Research indicates that both the gene and protein expression of Lp-PLA2 are significantly increased in the subcutaneous adipose tissue of patients with T2DM (55). *In vitro* studies further demonstrate that LDL or oxidized LDL significantly enhances Lp-PLA2 production in adipocytes, whereas inhibition of Lp-PLA2 leads to a reduction in oxidized LDL generation (55). These findings suggest that adipose tissue may be a primary source of Lp-PLA2 in states of obesity and T2DM, contributing to inflammation associated with lipid metabolism disorders.

Furthermore, clinical studies have identified a strong link between elevated Lp-PLA2 levels and diabetic microvascular complications. A cohort study conducted in the UK found that patients in the highest quartile of plasma Lp-PLA2 activity had more than twice the risk of developing diabetic retinopathy compared to those in the lowest quartile (79). In Chinese populations with T2DM, levels of Lp-PLA2 were significantly elevated in patients exhibiting early diabetic nephropathy, specifically at the microalbuminuria stage, compared to those without renal impairment (80). As a diagnostic marker for early nephropathy, Lp-PLA2 achieved an area under the ROC curve (AUC) of 0.841 (80). Additionally, research indicates that patients with diabetic peripheral neuropathy exhibit higher concentrations of Lp-PLA2, which is independently associated with an increased risk of neuropathy (odds ratio [OR] ≈ 1.011 (81). These findings suggest that Lp-PLA2 may serve as a promising biomarker for diabetic microvascular complications, including retinopathy, nephropathy, and neuropathy.

From a therapeutic perspective, Lp-PLA2 inhibitors exhibit promising clinical potential. A study involving patients with T2DM demonstrated that the Lp-PLA2 inhibitor, darapladib, significantly decreased the risk of major coronary events by 33% in individuals with inadequate glycemic control (82), This finding underscores the need for further investigation into the benefits of Lp-PLA2 inhibition in high-risk diabetic populations. In conclusion, Lp-PLA2 is integral to the inflammatory processes associated with diabetes and its complications, and its potential as both a diagnostic biomarker and a therapeutic target merits additional research. These disease-specific mechanisms align with the integrated pathological framework summarized in **Figure 4**, highlighting shared cytokine-driven inflammatory circuits across multiple disorders.

#### 2.2.5 Cancer

Extensive research has demonstrated that Lp-PLA2 is highly expressed in a variety of malignant tumors, with its expression levels significantly associated with increased tumor invasiveness and poor prognosis. It has been identified as a potential prognostic biomarker for several cancers (83–85). Pecifically, in bladder cancer, Lp-PLA2 expression is significantly elevated and inversely correlated with patient survival rates (13). Further mechanistic studies have elucidated that Lp-PLA2 can upregulate PD-L1 expression through activation of the JAK-STAT signaling pathway, thereby inhibiting anti-tumor thymus-derived lymphocyte (T-cell) immune responses and facilitating tumor immune evasion (13). Genome-wide analyses further suggest that Lp-PLA2 may serve as a prognostic marker in tumors such as prostate cancer, diffuse large B-cell lymphoma, and melanoma, where its elevated expression is closely linked to disease progression (83). Functional experiments have elucidated that Lp-PLA2 facilitates epithelial-mesenchymal transition (EMT) and enhances migratory capabilities in tumor cells, consequently expediting metastatic processes. In the context of immunoregulation, research suggests that Lp-PLA2 fosters the progression of hepatocellular carcinoma through the STAT1/PD-L1 signaling pathway. Furthermore, elevated expression of PLA2G7 in macrophages has been shown to inhibit CD8^+^ T cell activity, thereby intensifying tumor-associated immunosuppression (13).

Numerous studies have investigated the *in vivo* and *in vitro* roles of Lp-PLA2 within specific tumor models. Vainio et al. (19), demonstrated that Lp-PLA2 overexpression is prevalent in clinical prostate cancer tissues, with a significant positive correlation (r = 0.66, P < 0.001) observed between its expression and ERG, a member of the E26 transformation-specific family implicated in the initiation and progression of prostate cancer. Functional assays revealed that knockdown of Lp-PLA2 inhibited the proliferation of prostate cancer cells, induced apoptosis, and decreased migration; moreover, synergistic antiproliferative effects were noted when combined with statin treatment. Alinezhad et al. (86), further identified Lp-PLA2 as a potential biomarker for prostate cancer and assessed its role in disease progression. This study similarly reported that silencing Lp-PLA2 diminished tumor cell invasion and proliferation capacity. Additionally, Lp-PLA2 gene expression was significantly associated with phospholipid metabolic reprogramming in prostate cancer, indicating a potential pivotal role in pathogenesis.

In addition to the cancers previously discussed, several other malignancies have also demonstrated the involvement of Lp-PLA2, suggesting a degree of mechanistic convergence across tumor types. In bladder cancer, dysregulated phospholipid metabolism and inflammation-associated pathways have been implicated in tumor progression, and similar lipid-inflammation axes have been reported in other solid tumor (21). In prostate cancer, elevated expression of PLA2G7—the gene encoding Lp-PLA2—has been detected in tumor tissues, where it has been associated with cancer progression and may influence EMT and metastatic behavior (87). Notably, both bladder and prostate cancers exhibit increased levels of oxidized phospholipids and inflammatory mediators, aligning with observations in colorectal and breast cancers. In melanoma, although direct studies on Lp-PLA2 remain limited, lipidomic analyses and PLA2-related investigations have revealed substantial alterations in lysophospholipid metabolism and phospholipase-driven inflammatory remodeling, which may contribute to immune evasion and metastatic dissemination. These mechanisms are consistent with the broader lipid-remodeling functions of Lp-PLA2 described across multiple cancer types, including ferroptosis sensitization and membrane phospholipid restructuring (21). Recent reviews further highlight that Lp-PLA2 participates in cancer-associated inflammatory and metabolic pathways—such as NF-κB activation, oxidative lipid signaling, and EMT regulation—suggesting potential biological parallels among prostate, bladder, melanoma, breast, and colorectal cancers (88).Collectively, these findings suggest that Lp-PLA2 may serve as a valuable biomarker or therapeutic target, particularly in ERG-positive prostate cancer (86). Elevated expression of Lp-PLA2 serves as a diagnostic marker for identifying patients with highly invasive and metastatic tumors. Therapeutically, Lp-PLA2 inhibitors have shown potential in antitumor applications. Research indicates that the inhibitor darapladib enhances the sensitivity of cancer cells to ferroptosis, thereby inhibiting growth across various tumor types. In renal cell carcinoma cell lines, inhibition of Lp-PLA2 significantly reduced cellular viability (83). Further studies reveal that Lp-PLA2 exhibits variable alteration frequencies across human cancers and is correlated with pan-cancer tumor mutational burden, tumor microenvironment, DNA/RNA stemness scores, tumorigenesis, immune status, and microsatellite instability. Immunofluorescence and Western blot analyses have demonstrated high cytoplasmic expression of PLA2G7 in renal carcinoma cell lines, such as ACHN and 786-O. Treatment with the Lp-PLA2 inhibitor darapladib significantly suppressed cell viability in these lines (83). Additionally, animal studies have shown that combining Lp-PLA2 inhibition with immune checkpoint inhibitors, such as anti-CTLA-4 antibodies, synergistically reduces tumor burden and prolongs survival (13). In summary, Lp-PLA2 serves not only as a prognostic biomarker for multiple cancers but also holds promise as an emerging target for tumor immunotherapy and targeted treatment. As shown in **Figure 4**, cancers with elevated Lp-PLA2 expression converge on inflammatory mediators such as interleukin-6(IL-6), TNF-α, and ROS, consistent with the cross-tumor comparison presented in this section.

#### 2.2.6 Other diseases

In recent years, the potential of Lp-PLA2 as a prognostic or diagnostic biomarker has been extensively investigated across a range of diseases. Notably, in conditions where definitive biomarkers are lacking, such as Rett syndrome and autism spectrum disorder (ASD), serum levels of Lp-PLA2 in individuals with ASD have demonstrated a significant negative correlation when compared to healthy controls, thereby suggesting its utility as a novel biomarker (89). Furthermore, in patients with obstructive sleep apnea (OSA) and obesity, elevated levels of Lp-PLA2 have been associated with endothelial dysfunction. The reduction of this marker following OSA treatment indicates that plasma Lp-PLA2 activity may serve as a reliable indicator of atherosclerosis and vascular dysfunction risk in pediatric patients with obesity or OSA (83). In individuals diagnosed with HIV, Lp-PLA2 levels are significantly elevated, and these levels further increase upon the commencement of antiretroviral therapy (ART) or protease inhibitor treatment regimens. Lp-PLA2 is linked to various risk factors for HIV-associated cardiovascular disease (CVD) (90, 91), including carotid intima-media thickness (cIMT) and coronary artery calcification (CAC) (92). The administration of statins in individuals infected with HIV has been demonstrated to lower Lp-PLA2 levels, concurrently diminishing markers of immune activation and arterial inflammation. This intervention also reduces noncalcified plaque volume and improves the characteristics of high-risk coronary plaques (93). Consequently, Lp-PLA2 may not only serve as a potential predictor of subclinical atherosclerosis but also as a therapeutic target for the prevention of CVD in HIV-infected individuals. However, its clinical utility necessitates further validation (94).

Furthermore, elevated plasma Lp-PLA2 activity is commonly observed in antiphospholipid Antibody-positive patients indicate the potential of Lp-PLA2 as a prognostic biomarker for antiphospholipid syndrome (95). In individuals with rheumatoid arthritis (RA), baseline levels of Lp-PLA2 mass exhibit a significant correlation with disease severity and markers of subclinical atherosclerosis, such as carotid intima-media thickness and flow-mediated dilation. This correlation underscores its potential utility as a biomarker for vascular injury in RA (96). A prospective longitudinal cohort study further demonstrated that plasma Lp-PLA2 activity and mass are independently and significantly associated with the development of abdominal aortic aneurysm (AAA), highlighting its potential as a predictor of AAA risk (97). The preceding detailed studies highlight the extensive involvement of Lp-PLA2 in multiple pathological conditions. To integrate these findings and provide a comparative perspective, we summarize the key mechanisms, immune/lipid interactions, and levels of evidence for Lp-PLA2 in these diseases in **Table 2**. This systematic summary underscores the central role of Lp-PLA2 at the intersection of lipid metabolism and inflammatory signaling.

**Table 2:** Roles of Lp-PLA2 across diseases: mechanisms, immune/lipid interactions, evidence levels, and references.

Disease/Condition	Major mechanisms of Lp-PLA2	Key immune/Lipid interactions	Evidence level	References
Atherosclerosis/ASCVD	Hydrolyzes oxLDL → LysoPC/oxNEFA → endothelial dysfunction & plaque instability	LysoPC activates NADP-H oxidase; promotes M1- macrophages; impairs e-NOS coupling	Strong clinical evidence (cohorts + predictive studies + Phase III trials)	(38, 41–44, 47–51)
ACS/HF risk prediction	Elevated Lp-PLA2 reflects plaque vulnerability and inflammation	Synergy with CRP, IL-6 pathways	Clinical observational/predictive evidence	(46, 54)
Diabetes/Metabolic Syndrome	Promotes inflammatory lipid metabolism; correlates with dyslipidemia	Links adipose inflammation (IL-6, TNF-α) and lipid remodeling	Clinical evidence	(55, 117)
NASH	Inhibits JAK2/STAT3 → improves autophagy & inflammation	Shifts macrophage polarization; regulates fatty acid metabolism	Preclinical evidence	(56)
Alzheimer’s disease/Neurodegeneration	Disrupts BBB; promotes neuroinflammation	LysoPC activation of microglia	Primarily preclinical	(37)
Cancer (HCC, bladder, prostate)	High in TAMs; support-s immune evasion; inhibition enhances immune-therapy	Suppresses CD8^+^ T cells; regulates PD-L1 via J-AK-STAT axis	Preclinical + associative clinical studies	(10, 13, 83–88)
Autoimmune diseases (e.g., Sjögren’s lymphoma)	Modulates DC/T cell activation	Influences autoreactive lymphocyte responses	Clinical association evidence	(93)
HIV-associated inflammation/CVD risk	Elevated during ART; increases vascular inflammation	Links to cIMT, CAC, T-NF-α activation	Clinical observational evidence	(90–92)
COPD	Correlates with airway inflammation & exacerbations	Lipid mediators recruit-t neutrophils	Clinical observational evidence	(57)
Obstructive sleep apnea (OSA)	Elevated Lp-PLA2 in children → endothelial dysfunction	LysoPC-mediated endothelial activation	Clinical observational evidence	(95)
Diabetic retinal vasopermeability	Lp-PLA2 increases vascular leakage; inhibitors reduce permeability	LysoPC enhances endothelial permeability	Preclinical + small clinical studies	(12)

## 3 Lp-PLA2 and immunity

Lp-PLA2 functions at the interface of lipid metabolism, inflammation, and immunity, and its expression is closely regulated by pro-inflammatory and immune-related signals. Pro-inflammatory cytokines such as TNF-α, IL-6, and interferon gamma (IFN-γ) significantly enhance its transcription and secretion, with NF-κB and PPARγ serving as critical transcriptional regulators. Metabolic factors, including high-fat diets and oxidative stress, further augment its enzymatic activity. The hydrolytic products of Lp-PLA2, including LysoPC and free fatty acids (FFA), play essential roles in inflammatory and immune signaling. LysoPC induces endothelial cells to overexpress adhesion molecules: vascular cell adhesion molecule-1 (VCAM-1)、intercellular adhesion molecule-1 (ICAM-1) and monocyte chemoattractant protein-1 (MCP-1), promoting monocyte adhesion and infiltration. It also stimulates macrophages to produce ROS and cytokines such as TNF-α and IL-1β, intensifying local inflammatory responses (4). LysoPC can induce endothelial cells to express adhesion molecules and chemokines, promote immune cell recruitment, and stimulate macrophages to produce proinflammatory mediators. FFA activate the Toll-like receptor 4/nuclear factor κB (TLR4/NF-κB) signaling pathway, promote the release of proinflammatory mediators, and participate in lipid peroxidation and apoptosis processes (98). It is worth noting that LysoPC may function as an intracellular second messenger involved in gene regulation (99), and Lp-PLA2-mediated generation of non-esterified fatty acids adds another layer of inflammatory modulation.

Substantial evidence suggests that Lp-PLA2 is pivotal in vascular inflammation in atherosclerosis. Macrophage-derived Lp-PLA2 hydrolyzes oxidized LDL to produce LysoPC and FFA, contributing to foam cell formation and plaque instability. Elevated circulating Lp-PLA2 levels are associated with an increased risk of cardiovascular events, making it a critical biomarker for atherosclerotic progression (100). However, the biological role of Lp-PLA2 is multifaceted: it can degrade PAF, exerting anti-inflammatory effects (101), while also generating pro-inflammatory mediators that exacerbate lipid-driven inflammation (98). Notably, LysoPC can also be generated non-enzymatically through spontaneous deacylation of oxidized phospholipids under physiological conditions (102), a process not inhibited by antiphospholipase drugs, underscoring the need for novel therapeutic strategies.

### 3.1 Lp-PLA2 and immune cell function

Lp-PLA2, also referred to as platelet-activating factor acyl hydrolase, has attracted considerable scholarly interest in recent years as a factor associated with inflammation. This enzyme is predominantly synthesized and secreted by the monocyte-macrophage system, with its encoding gene demonstrating elevated expression levels in macrophages (34, 103). Lp-PLA2 is primarily localized in tissue macrophages, including those associated with neoplastic tissues. Its enzymatic products, such as LysoPC, have the capacity to activate immunoregulatory receptors, including the G2 Accumulation Receptor (G2A) or G Protein-Coupled Receptor 132 (GPR132). This receptor activation facilitates the polarization of macrophages toward the pro-inflammatory M1 phenotype (104).

This process is chiefly mediated by oxLDL and LysoPC and can be reversed through the application of Lp-PLA2 inhibitors. Experimental investigations have revealed altered macrophage polarization in Lp-PLA2-deficient mice, characterized by a significant reduction in M1 macrophages and an increase in regulatory M2 macrophages, accompanied by diminished systemic inflammation and lower peripheral blood concentrations of IL-6 and interleukin-12(IL-12) (104). These findings suggest that Lp-PLA2 plays a critical role in the inflammatory response by modulating macrophage M1/M2 differentiation. In alignment with this, under conditions of heightened inflammation, such as those observed in atherosclerosis, LysoPC produced by the combined action of Lp-PLA2 and oxLDL further activates macrophages and T lymphocytes. This activation results in an increased release of inflammatory mediators, thereby establishing a positive feedback loop (104). Research also suggests that the promotion of M1 macrophage polarization by Lp-PLA2 can be mitigated by inhibitors like daliprazine, indicating that this process is primarily mediated through the oxLDL/LysoPC pathway. Additionally, single-cell sequencing data demonstrate that in tumor microenvironments, such as hepatocellular carcinoma, Lp-PLA2 is predominantly expressed in tumor-associated macrophages (10), and is closely linked to local immune suppression. Macrophages with elevated Lp-PLA2 expression inhibit CD8^+^ T cell activity, thereby facilitating tumor immune evasion. Lipid rafts are cholesterol- and sphingomyelin-rich microdomains on the cell membrane that concentrate TCRs, co-stimulatory molecules, and downstream kinases, directly participating in signal transduction and immunological synapse formation in T cells and dendritic cells (DCs). For example, in T cells from CLL patients, membrane cholesterol deficiency leads to reduced lipid rafts, impaired TCR clustering, and diminished immune function (105). Conversely, cholesterol reverse transport mediated by ABCA1/ABCG1 or HDL processing can disrupt antigen-presenting cell membrane structures, suppress DC-mediated T cell activation, and promote immune tolerance. In DCs, enhanced fatty acid synthesis (upregulated FAS activity) promotes lipid droplet formation and enhances major histocompatibility complex (MHC) class I/II antigen cross-presentation capacity (106); Excessive lipid accumulation (particularly oxidized lipids) impairs their function: DCs in tumors or high-fat environments are rich in oxidized triglycerides and oxidized phospholipids, significantly reducing surface MHC-antigen complex expression and suppressing T cell activation (106, 107). Additionally, endogenous lipid metabolites such as LPC and oxFA serve as activation signals for the NLRP3 inflammasome. Experimental studies have demonstrated that LPC induces NLRP3 inflammasome assembly and IL-1β secretion in both macrophages and endothelial cells; Certain saturated fatty acids and their derivatives (such as palmitic acid derivatives or sphingomyelin metabolites) can also amplify proinflammatory signaling by promoting the NLRP3/caspase-1 pathway (69). Lp-PLA2 is secreted by macrophages and other cells, hydrolyzing oxidized phospholipids to generate LysoPC and oxFA (108), These products can alter the lipid composition of immune cell membranes, disrupting lipid raft stability and receptor clustering, while also acting as “danger signals” to amplify inflammation: LysoPC not only induces lipid droplet formation but also activates NLRP3 inflammasomes to trigger inflammatory pyroptosis (69), oxFA promotes the inflammatory cascade through pathways such as TLR signaling and lysosomal rupture (109). In summary, lipid raft-mediated membrane signaling, the lipid metabolic state of dendritic cells (DCs), and NLRP3 inflammasome signaling constitute an interconnected network. Within this network, Lp-PLA2 contributes by generating inflammatory lipid metabolites, collectively modulating both innate and adaptive immune responses (105, 106).

**Figure 6:**
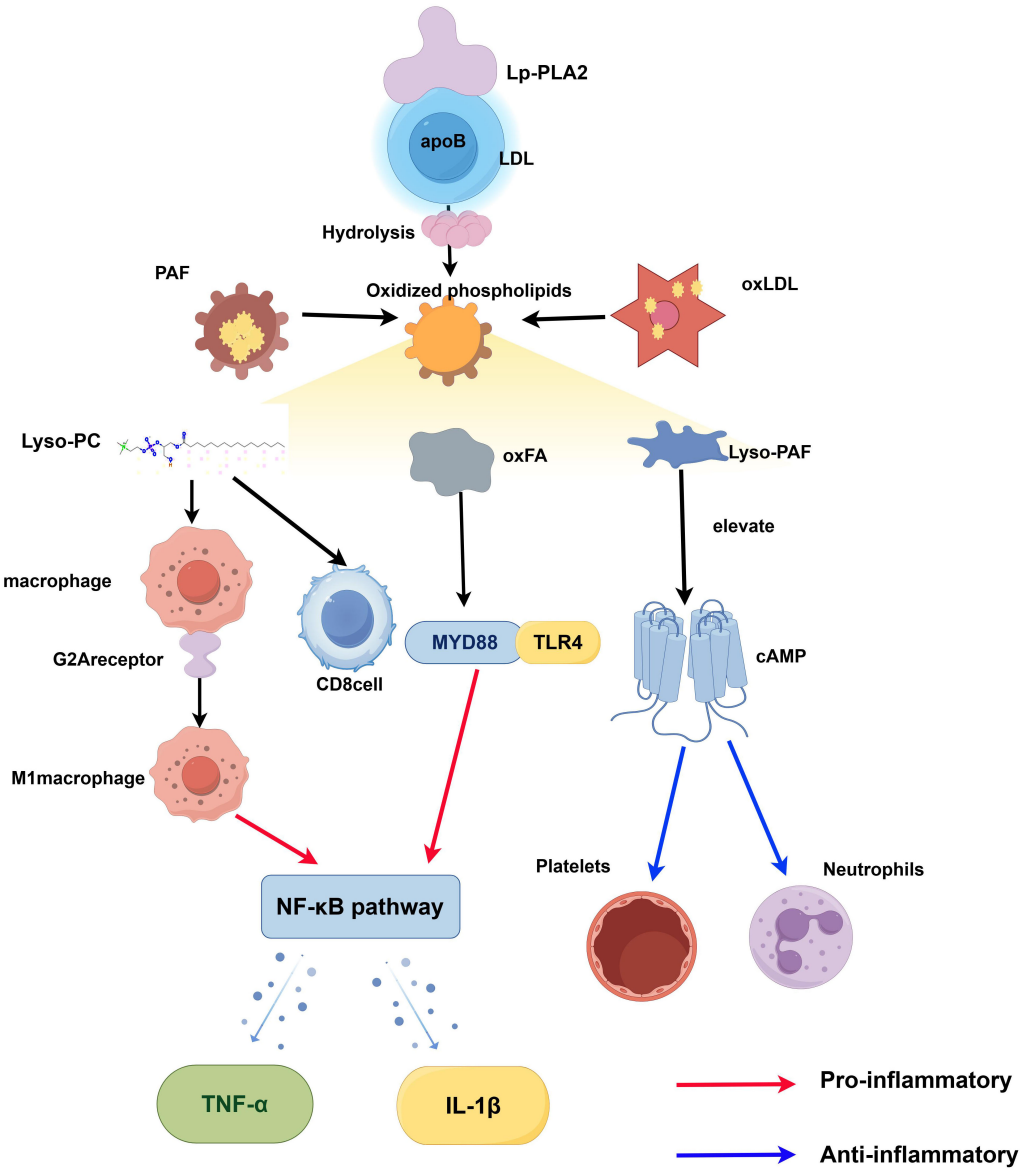
The mechanistic model of Lp-PLA2 in immunoregulation involves its binding to LDL, where it hydrolyzes oxidized phospholipids and PAF to produce LysoPC, LysoPS, and oxFA. LysoPC activates G2A receptors on macrophages, promoting M1 polarization and the release of TNF-α and IL-1β, while inhibiting CD8^+^ T cell activation. LysoPAF has an anti-inflammatory effect by increasing cAMP levels, which inhibits neutrophil and platelet activity. Red arrows indicate pro-inflammatory pathways, while blue arrows represent anti-inflammatory or inhibitory pathways.

Consequently, Lp-PLA2 assumes a pivotal immunoregulatory role in macrophages by influencing polarization states and inflammatory pathways, thereby modulating immune cell function and presenting itself as a promising therapeutic target. The core regulatory mechanisms of Lp-PLA2 in immune cells, particularly the dual role of its hydrolysates in macrophage polarization and T cell function, merit further investigation and are systematically summarized in **Figure 6**.

### 3.2 Lp-PLA2 and autoimmune diseases

#### 3.2.1 Systemic lupus erythematosus

The expression of Lp-PLA2 is often upregulated in a variety of autoimmune diseases and is closely linked to disease activity. Recent research has demonstrated that serum Lp-PLA2 activity in patients with systemic lupus erythematosus (SLE) is markedly elevated compared to healthy controls (221 ± 56 U/L vs. 160 ± 37 U/L, p < 0.001), with particularly high levels observed in patients exhibiting clinical manifestations such as lupus nephritis and anemia (93). The same study suggested that serum Lp-PLA2 activity could serve as a novel biomarker for B-cell proliferation and a potential therapeutic target in patients with primary Sjögren’s syndrome (SS) (93). In a study comprising 154 SLE patients and 55 healthy controls matched for age, sex, and body mass index, serum Lp-PLA2 activity was measured alongside anthropometric parameters, clinical manifestations, SLEDAI-2K scores, complement C3 and C4 levels, erythrocyte sedimentation rate (ESR), and autoantibodies. The findings revealed that the mean serum Lp-PLA2 activity in the SLE cohort was 221 ± 56 U/L, significantly surpassing the 160 ± 37 U/L observed in the control group (p < 0.001. In patients with nephritis, anemia, or fibrinolytic abnormalities, Lp-PLA2 activity was significantly elevated compared to those without these conditions (p < 0.05), and this activity demonstrated a positive correlation with clinical severity (p < 0.001). No significant correlation was observed between serum Lp-PLA2 activity and autoantibody levels (p > 0.05). Spearman correlation analysis revealed significant associations between Lp-PLA2 activity and ESR, SLEDAI-2K, C3, and C4 levels (p < 0.001). Furthermore, binary logistic regression analysis identified Lp-PLA2 activity as an independent risk factor for active systemic lupus erythematosus (SLE) (OR = 1.049, 95% CI: 1.025–1.073, p < 0.001). These findings suggest a potential link between Lp-PLA2 activity and specific clinical manifestations of SLE, such as nephritis, anemia, and fibrinolytic abnormalities, as well as its possible role in disease progression.

Lp-PLA2 activity demonstrated significant positive correlations with the systemic lupus erythematosus disease activity index 2000(SLEDAI-2K), complement levels, and various inflammatory markers. Multivariate analyses further corroborated its role as an independent risk factor for SLE activity, indicating that Lp-PLA2 may be involved in the pathological mechanisms underlying autoimmune diseases such as lupus.

Animal studies further substantiate the pro-inflammatory role of Lp-PLA2 in autoimmunity; specifically, in the experimental autoimmune encephalomyelitis (EAE) model, which serves as a murine analog of multiple sclerosis, Lp-PLA2 facilitates macrophage polarization toward the M1 phenotype through the oxLDL and LysoPC pathways, thereby augmenting inflammatory responses (104). Inhibition of Lp-PLA2, for instance, via the small-molecule inhibitor darapladib, has been shown to reduce the release of inflammatory mediators, suppress AngII-induced NLRP3 inflammasome activation, and consequently mitigate central nervous system inflammation (104). In conclusion, Lp-PLA2 is integral to the pathogenesis and progression of autoimmune diseases such as SLE and EAE, primarily through its regulation of macrophage-mediated inflammatory signaling pathways. At present, therapeutic strategies focusing on Lp-PLA2 are receiving considerable scholarly attention. This includes investigating the potential application of Lp-PLA2 inhibitors and biologics in mitigating autoimmune inflammation and enhancing patient prognosis.

#### 3.2.2 Rheumatoid arthritis and Sjögren’s syndrome

Studies indicate that Lp-PLA2 expression differs in rheumatoid arthritis (RA) and sjögren’s syndrome (SS) and may relate to clinical severity. In early RA, circulating Lp-PLA2 activity is actually reduced: Evangelia Lourida et al. found that patients with recent-onset RA had significantly lower total plasma and HDL-associated Lp-PLA2 activity than healthy controls (110). Notably, one year of antirheumatic therapy (methotrexate/prednisone) normalized HDL−associated Lp-PLA2 activity as inflammation subsided. In contrast, Lp-PLA2 has generally not been reported as a direct marker of RA activity-rather, it has been studied as a link to RA-associated atherosclerosis (111). In primary Sjögren’s syndrome, Lp-PLA2 levels appear elevated. A recent Turkish study found higher median serum Lp-PLA2 in primary sjögren’s syndrome (pSS) patients than controls (560 vs. 328 ng/mL, p=0.024). and levels correlated inversely with ESR (r=-0.409). Crucially, Lp-PLA2 is markedly upregulated in SS patients who develop lymphoma: serum Lp-PLA2 activity (and salivary-gland Lp-PLA2 expression) was significantly higher in SS patients with B-cell lymphoma than in those without or healthy controls (93). No significant Lp-PLA2 difference was seen between lymphoma-free SS and healthy individuals, suggesting the rise is linked to lymphoma risk rather than basal disease.

Direct studies of Lp-PLA2 in RA/SS models are limited, but preclinical data from related inflammation models suggest therapeutic promise. In cardiovascular models, Lp-PLA2 inhibitors like darapladib have demonstrated anti-inflammatory effects: e.g. in AngII-infused mice, darapladib reduced macrophage migration, IL-1β release and cardiac fibrosis by blocking NLRP3 activation (108). In autoimmune models, PLA2G7-deficient mice are protected from inflammation and metabolic stress. For instance, Spadaro et al. reported that PlA2G7 knockout mice had lower systemic IL-1β, IL-6 and IL-12 levels and far fewer inflammatory (M1) macrophages after LPS/ceramide challenge (57, 104). Notably, these mice showed a shift to regulatory (M2) macrophages and preserved thymic function, highlighting that Lp-PLA2 loss broadly attenuates inflammatory pathways and even improves T-cell generation (104). Although no Lp-PLA2 inhibitors have been tested specifically in RA/SS, existing data imply they could modulate disease. In early RA patients, standard treatment raised HDL–Lp-PLA2 activity as inflammation decreased (110). By extension, an Lp-PLA2 inhibitor might have a similar anti-inflammatory effect. In SS, the finding that Lp-PLA2 drives B-cell lymphoproliferation suggests targeting it could impede lymphoma development (93). In fact, authors from Frontiers suggest that Lp-PLA2 may serve as “a novel therapeutic target for B-cell lymphoproliferation” in Sjögren’s Syndrome (SS). Beyond its implications in autoimmunity, current clinical trials in atherosclerosis and cancer, along with animal studies on Lp-PLA2 inhibition, further substantiate its potential to mitigate inflammation and abnormal immune activation.

Lp-PLA2 has attracted attention as a biomarker in both RA and SS, though evidence differs between diseases. In RA, most work frames Lp-PLA2 as a marker of accelerated atherosclerosis rather than of arthritis itself. For example, RA cohorts have shown associations of Lp-PLA2 with subclinical vascular lesions, reflecting systemic inflammation (111, 112). However, because early RA patients actually have low Lp-PLA2 activity (110). it has not emerged as a diagnostic RA marker. By contrast, in SS Lp-PLA2 shows clearer biomarker value. Serum Lp-PLA2 activity is higher in pSS patients than in controls. suggesting it could flag cardiovascular risk in SS. More strikingly, Lp-PLA2 activity (and glandular expression) was significantly elevated in SS patients with lymphoma (93). The ROC analysis from that study indicated that serum Lp-PLA2 could differentiate between SS patients with and without lymphoma (AUC ~0.80). Therefore, Lp-PLA2 is being studied as a potential prognostic biomarker for lymphoma development in SS. In conclusion, although Lp-PLA2 is not recognized as a marker for RA, it is being investigated in SS, particularly as a sign of malignant B-cell growth (93).

### 3.3 Lp-PLA2 and infection-mediated immunity

During immune responses to infection and sepsis Lp-PLA2 exhibits proinflammatory effects and is considered a potential target for modulating immune reactions. Both *in vivo* and *in vitro* studies have demonstrated that stimulation with bacterial endotoxin (lipopolysaccharide, LPS) significantly enhances the transcription and secretion of Lp-PLA2 in macrophages (103). This upregulation is closely linked to increased levels of proinflammatory cytokines, including tumor necrosis TNF-α, IL-1β, and IL-6, thereby exacerbating inflammatory damage. In RAW264.7 macrophages treated with LPS, Lp-PLA2 expression increased concomitantly with the release of inflammatory mediators. Conversely, the overexpression of microRNA-494-3p, which suppresses Lp-PLA2, significantly reduced the secretion of these cytokines and alleviated cellular inflammatory responses (103). Elevated serum levels of Lp-PLA2 were similarly observed in a mouse model of sepsis, showing a positive correlation with markers of inflammatory injury (95). Mechanistic investigations revealed that the transcription factor Spi1 activates Lp-PLA2 expression, thereby promoting the production of proinflammatory cytokines; the knockdown of either Lp-PLA2 or Spi1 mitigated cytokine storms and organ damage in septic mice (113). Moreover, Lp-PLA2 inhibitors exhibit promising therapeutic potential in the context of infection-related immunotherapy. In a cardiac inflammation model induced by angiotensin II (Ang II), the inhibition of Lp-PLA2 effectively suppressed the activation of the macrophage NLRP3 inflammasome (57, 104). In the case of viral infections, such as COVID-19, elevated Lp-PLA2 levels are regarded as a potential inflammatory biomarker for evaluating the risk of severe disease (114). Overall, Lp-PLA2 plays a role in exacerbating inflammatory responses during infections, and its inhibition leads to a reduction in the release of inflammatory mediators. This indicates that targeting Lp-PLA2 may offer immunomodulatory benefits for the treatment of infectious diseases (103).

### 3.4 Lp-PLA2 and immunometabolism

Lp-PLA2 has been identified as a pivotal gene in the regulation of immunometabolic processes within adipose tissue metabolism (57, 115). Research indicates that sustained moderate calorie restriction (14%) leads to a significant downregulation of Lp-PLA2 expression, which is associated with decreased systemic inflammation and increased lifespan (104). In alignment with these findings, Lp-PLA2-deficient mice display an anti-obesity phenotype when subjected to a high-fat diet, characterized by decelerated weight gain, diminished hepatic steatosis, and elevated levels of glycerol and free fatty acids in adipose tissue, indicative of enhanced lipolysis. These metabolic alterations are accompanied by a reprogramming of macrophage phenotypes, evidenced by a reduction in classical pro-inflammatory M1 macrophages and an increase in regulatory M2 macrophages in Lp-PLA2-deficient mice. Given that M2 macrophages are primarily involved in tissue repair and oxidative metabolism, this phenotypic shift suggests that Lp-PLA2 plays a role in modulating metabolic pathways within immune cells. Furthermore, Lp-PLA2 knockout models exhibit reduced NLRP3 inflammasome signaling, increased thymus volume, and a higher number of thymocytes, indicating a delay in the aging of immune organs. These findings collectively illustrate that Lp-PLA2 functions as a pivotal regulator at the intersection of lipid metabolism and immune function. Inhibition of Lp-PLA2 replicates the immunometabolic effects associated with calorie restriction (CR), enhancing energy metabolism while concurrently suppressing inflammatory pathways. As a result, Lp-PLA2 has attracted considerable interest within the field of immunometabolism. Ongoing research continues to elucidate its regulatory mechanisms and assess its potential as a therapeutic target for metabolic inflammatory diseases, such as obesity and diabetes (104).

Further investigations have uncovered additional roles of Lp-PLA2 within the immune-metabolic interaction network, including its involvement in the regulation of thymic senescence, adipose tissue inflammation, metabolic syndrome, and inflammasome pathways. This gene has been identified as a central molecule in immune-metabolic regulation, with its expression closely associated with inflammatory responses and aging processes. Sustained moderate calorie restriction significantly reduces Lp-PLA2 expression, which is accompanied by decreased systemic inflammation and an extended healthy lifespan (116). In a two-year clinical trial involving a 14% reduction in caloric intake, a downregulation of Lp-PLA2 was associated with enhanced thymic function, as evidenced by increased T cell proliferation, and a decrease in inflammation within adipose tissue. Similarly, Lp-PLA2 knockout mice demonstrated anti-obesity and metabolically protective effects when subjected to a high-fat diet, including reduced weight gain, decreased hepatic lipid accumulation, and increased levels of glycerol and free fatty acids in adipose tissue, indicative of enhanced lipolysis (104). Clinical studies further underscore a significant correlation between elevated Lp-PLA2 levels and an increased risk of metabolic syndrome (117). Collectively, these findings suggest that Lp-PLA2 plays a role in maintaining energy homeostasis by modulating adipose tissue metabolism and inflammatory pathways. Inhibition of Lp-PLA2 function appears to replicate the beneficial effects of caloric restriction, thereby improving energy metabolism and mitigating metabolic inflammation.

Mechanistic investigations reveal that the knockout of Lp-PLA2 facilitates the polarization of macrophages toward the M2 anti-inflammatory and repair phenotype, diminishes the prevalence of classical M1 pro-inflammatory macrophages, and reduces systemic inflammatory mediators such as IL-6 and IL-12 (104). Simultaneously, the absence of Lp-PLA2 markedly suppresses the activation of the NLRP3 inflammasome; upon stimulation with LPS or ceramide, the activation levels of inflammasome markers are diminished in macrophages lacking Lp-PLA2. Moreover, 24-month-old knockout mice display enlarged thymuses with an increased number of thymocytes, suggesting protective effects against age-associated thymic atrophy (104). Additionally, LysoPC catalyzed by Lp-PLA2, modulates T lymphocyte chemotaxis and metabolic processes through G2A receptors, thereby affecting T cell effector functions (118). In conclusion, Lp-PLA2 plays a complex and multi-dimensional role in immune regulation, encompassing binding to lipoproteins, exerting both pro-inflammatory and anti-inflammatory effects, modulating inflammasome activity, and interacting with immune metabolism.

## 4 Clinical significance and therapeutic strategies

Recent advances have highlighted the clinical relevance of Lp-PLA2 across metabolic, immune-mediated, and neoplastic diseases, establishing it as a promising biomarker and therapeutic target beyond the cardiovascular system. In oncology and immunology, Lp-PLA2 is predominantly overexpressed in tumor-associated macrophages in cancers such as hepatocellular carcinoma and bladder cancer, where it contributes to immune evasion and unfavorable prognosis (10). Inhibition of Lp-PLA2 enhances CD8^+^ T-cell activation and improves the responsiveness to immune checkpoint blockade, underscoring its potential as an immunotherapeutic target. Similarly, in EAE, pharmacological inhibition of Lp-PLA2 reduces M1 macrophage polarization and attenuates neuroinflammation and demyelination (115). Genetic polymorphisms of PLA2G7 have also been linked to autoimmune disorders such as systemic lupus erythematosus, ulcerative colitis, and Kawasaki disease (114, 119), suggesting its potential value as an indicator of disease susceptibility and activity.

Therapeutic strategies targeting Lp-PLA2 have primarily focused on small-molecule inhibition. Although early-generation inhibitors such as darapladib showed promising anti-inflammatory effects in preclinical models, clinical benefits were limited in large cardiovascular outcome trials. Nevertheless, subgroup analyses and mechanistic studies suggest that specific patient populations—such as individuals with diabetes or PLA2G7 high-activity genotypes—may derive benefit from Lp-PLA2 inhibition. Beyond cardiovascular disease, emerging evidence indicates potential therapeutic utility in microvascular and neuroinflammatory disorders, including diabetic macular edema and Alzheimer’s disease (5).

Drug discovery efforts have expanded to include fragment-based screening and natural-product-derived scaffolds. Several xanthine-based inhibitors, including AX10185 and its optimized derivatives, have demonstrated potent enzymatic inhibition *in vitro* (120). Natural compounds such as glyceride derivatives isolated from Cyperus rotundus also exhibit moderate inhibitory activity. Despite these advances, most inhibitors remain in preclinical development, with selectivity, bioavailability, and pharmacokinetic optimization representing major challenges for future translation.

Genetic and epigenetic approaches provide additional avenues for modulating Lp-PLA2 activity. Knockout or RNA interference–mediated suppression of Lp-PLA2 improves metabolic inflammation and reduces lipid accumulation in high-fat diet models (116, 121). In ApoE-deficient mice, RNA interference targeting PLA2G7 significantly attenuates plaque formation (3). Epigenetic analyses reveal hypomethylation in PLA2G7 promoter regions within atherosclerotic plaques, enhancing Sp3 binding and transcriptional activation (15). These findings support the feasibility of modulating Lp-PLA2 expression through epigenetic or gene-silencing strategies, although precision, safety, and delivery remain substantial obstacles.

From an immunotherapeutic perspective, Lp-PLA2 represents both an opportunity and a challenge. While its inhibition may dampen pro-inflammatory macrophage responses and enhance anti-tumor immunity, complete suppression could compromise host defense and disrupt lipid-mediated immune signaling. Patient stratification, genetic background, and disease-specific immune context are therefore critical for determining therapeutic suitability. Further translational and clinical studies are needed to establish safety, efficacy, and optimal therapeutic windows for Lp-PLA2–targeted interventions.

## 5 Translational relevance and clinical context

Two large Phase III trials (STABILITY and SOLID-TIMI 52) evaluating darapladib, an Lp-PLA2 inhibitor, in cardiovascular disease failed to demonstrate a significant reduction in the incidence of major cardiovascular events (122, 123). Analysis suggests this outcome relates to multiple factors including patient population and timing of intervention: most subjects were coronary heart disease patients already receiving standard therapies like statins, indicating very high baseline treatment levels and extremely low residual risk, which may have masked the modest benefit of Lp-PLA2 inhibition (123). The trials included heterogeneous populations (mixing stable patients with acute coronary syndrome patients) and lacked pre-specified screening for subgroups with high inflammatory burden or elevated Lp-PLA2 levels. Furthermore, genetic studies indicate that PLA2G7 gene inactivating variants do not significantly reduce coronary heart disease(CHD) risk, supporting the view that Lp-PLA2 functions more as an inflammatory marker than a key pathogenic target. In subgroup analyses, patients with conditions associated with high oxidative stress and inflammation, such as smoking, appeared potentially more responsive to Lp-PLA2 inhibition. However, overall, adding daliprazole to optimal therapy did not yield additional benefits (4).

Physiologically, Lp-PLA2 functions dualistically: primarily secreted by macrophages, it circulates bound to lipoproteins. When bound to ApoB-rich LDL, Lp-PLA2 hydrolyzes oxidized phospholipids to produce lysophosphatidylcholine and oxidized free fatty acids, the latter of which can trigger inflammatory responses in endothelial, smooth muscle, and immune cells. Conversely, when bound to ApoA-I-rich HDL, Lp-PLA2 is thought to exert anti-inflammatory and anti-atherosclerotic effects. Daliprazole’s systemic inhibition of Lp-PLA2 may simultaneously eliminate both these “harmful” and “beneficial” effects. The drug itself exhibits high selectivity with minimal impact on other PLA2 subtypes, but clinically reported side effects include gastrointestinal discomfort and body odor (with discontinuation in a small number of patients) (124). Moreover, studies utilizing animal models, such as ApoE^-^/^-^ mice, have shown that the inhibition of Lp-PLA2 can suppress the formation of atherosclerotic plaques. Nevertheless, these models fail to fully replicate the intricate pathology observed in humans, who often present with multiple risk factors, are frequently on concomitant statin therapy, and exhibit unique plaque characteristics and inflammatory states. As a result, the mechanisms observed in preclinical studies may not correspond directly to the outcomes observed in population-based interventions (4, 123).

Despite compelling preclinical efficacy, many anti-inflammatory targets have repeatedly failed in clinical translation. Several general obstacles contribute to this translational gap. First, poor predictive validity of animal models is a major issue: preclinical models often fail to recapitulate the complexity of human disease, including heterogeneity of immune activation, comorbidities, and temporal dynamics (125). Second, differences in experimental design—such as the timing of intervention and endpoint measurement—frequently bias toward early benefit in animal studies that cannot be reproduced in patients. For instance, meta-analyses in large animal models of myocardial infarction show that anti-inflammatory agents are most effective when assessed early after injury, a condition rarely mirrored in human trials (126). Third, regulatory and translational challenges remain: insufficient demonstration of target engagement in human tissues, lack of robust biomarkers to identify responsive subgroups, and a failure to stratify patients based on inflammation biology hamper clinical success (127). Finally, safety and off-target risks in human populations can be underestimated in preclinical testing, and regulatory pathways demand rigorous demonstration of both mechanism and clinical benefit (128). Collectively, these obstacles underscore why even well-validated inflammatory targets—such as Lp-PLA2—face formidable barriers in clinical translation. To improve success rates, future strategies must emphasize model refinement, biomarker-guided patient selection, early-phase proof-of-mechanism studies, and robust translational frameworks that bridge preclinical promise with human therapeutic potential.

## 6 Conclusion and outlook

Lp-PLA2 is a pivotal molecule situated at the intersection of lipid metabolism and immune response pathways, with its complex biological functions increasingly being elucidated in recent years. This enzyme plays a dual role: it hydrolyzes oxidized lipids, such as oxidized low-density lipoprotein, to produce bioactive mediators that contribute to vascular endothelial dysfunction and plaque formation. Concurrently, the cleavage products of Lp-PLA2 modulate inflammatory signaling pathways, including macrophage activation, the NLRP3 inflammasome, and the expression of proinflammatory cytokines (115, 116). Recent research further suggests that Lp-PLA2 serves not only as a pathological marker and regulatory factor in cardiovascular diseases (121), but also plays a significant role in processes such as tissue aging, metabolic homeostasis, and tumor immunity. In summary, Lp-PLA2 is centrally positioned at the convergence of lipid metabolism and immune responses, with its excessive activation being closely linked to the onset and progression of various diseases, including atherosclerosis, diabetic complications, neurodegenerative disorders, and tumor immune evasion (121, 129). Simultaneously, Lp-PLA2 demonstrates anti-inflammatory properties under specific conditions, underscoring the complexity of its dual functional nature.

Substantial evidence has accumulated supporting the pivotal role of Lp-PLA2 across various disease contexts. In the field of cardiovascular medicine, Lp-PLA2 functions as an inflammatory biomarker for predicting plaque stability and event risk, although the clinical efficacy of its inhibition remains inconclusive (4). Within models of metabolic syndrome and obesity, the knockout of Lp-PLA2 leads to increased energy expenditure and reduced fat accumulation, indicating its therapeutic potential for metabolic disorders (116). In autoimmune and inflammatory diseases, Lp-PLA2 is involved in pathological processes through the regulation of macrophage polarization (115). In the context of tumor immunity, elevated Lp-PLA2 expression is closely linked to immunosuppressive microenvironments (10), suggesting its potential as an adjunct target for immunotherapy. Consequently, Lp-PLA2 occupies a central position in multidisciplinary research. As a key molecule that bridges lipid metabolism and immune regulation, it holds significant implications for disease diagnosis, prognosis assessment, and the development of therapeutic strategies.

Future research should pursue several key directions. First, it is imperative to clarify the outstanding questions regarding the molecular mechanisms of Lp-PLA2, with an emphasis on elucidating the specific regulatory mechanisms that govern its dual roles—pro-inflammatory and protective—across diverse cell types and physiological conditions. This context-dependent behavior may arise from differences in the substrate microenvironment, local metabolic status, or co-activated signaling pathways, necessitating a comprehensive investigation using molecular biology techniques and animal models. Second, interdisciplinary collaboration should be enhanced by integrating methodologies from cardiovascular medicine, metabolic research, neuroscience, and tumor immunology. Employing systems biology, immunomics, and metabolomics strategies will facilitate a thorough analysis of Lp-PLA2’s functional networks and regulatory circuits. Third, efforts should be directed toward advancing the clinical validation of Lp-PLA2 as a biomarker. Large-scale prospective clinical trials are needed to elucidate its correlation with disease progression, establish standardized detection protocols, and assess its predictive value. Fourth, concerted efforts are necessary to advance the development of safer and more efficacious targeted therapeutics. In light of dapalizumab’s inability to meet expectations in large-scale clinical trials, it is imperative to actively investigate novel inhibitors and combination strategies. These may include small molecule-antibody conjugate therapies or the integration of Lp-PLA2 inhibition with other anti-inflammatory or metabolic modulators. Emerging technologies, such as CRISPR-Cas9 gene editing and RNA interference, hold considerable promise but necessitate the optimization of delivery systems and the resolution of off-target effects. Additionally, the influence of epigenetic regulation on Lp-PLA2 merits further investigation. Existing studies indicate that the methylation status of its promoter is associated with plaque stability (15). Future research should consider the modulation of Lp-PLA2 expression and activity through epigenetic drugs, such as DNA methylation inhibitors or specific miRNAs, thereby potentially intervening in downstream inflammatory responses.

In conclusion, Lp-PLA2 serves as a crucial mediator connecting lipid metabolism with immune system functions, and ongoing research continues to reveal its complex roles. Future research initiatives should integrate fundamental mechanistic investigations, clinical validation, and technological advancements to comprehensively evaluate its potential in disease prevention and treatment. Such an approach will lay a robust groundwork for the development of pertinent biomarkers and targeted therapies. Through these efforts, research on Lp-PLA2 is poised to contribute significantly to advancements in atherosclerosis, glucose metabolism disorders, autoimmune diseases, and tumor immunotherapy.
